# Adenosine-independent regulation of the sleep–wake cycle by astrocyte activity

**DOI:** 10.1038/s41421-022-00498-9

**Published:** 2023-02-07

**Authors:** Wanling Peng, Xiaotong Liu, Guofen Ma, Zhaofa Wu, Ziyue Wang, Xiang Fei, Meiling Qin, Lizhao Wang, Yulong Li, Siyu Zhang, Min Xu

**Affiliations:** 1 Institute of Neuroscience, State Key Laboratory of Neuroscience, CAS Center for Excellence in Brain Science and Intelligence Technology, Chinese Academy of Sciences, Shanghai, China; 2 University of Chinese Academy of Sciences, Beijing, China; 3 Center for Brain Science of Shanghai Children’s Medical Center, Department of Anatomy and Physiology, Shanghai Jiao Tong University School of Medicine, Shanghai, China; 4 Songjiang Institute and Songjiang Hospital, Shanghai Jiao Tong University School of Medicine, Shanghai, China; 5 State Key Laboratory of Membrane Biology, Peking University School of Life Sciences, Beijing, China; 6 Peking-Tsinghua Center for Life Sciences, Academy for Advanced Interdisciplinary Studies, Peking University, Beijing, China; 7 PKU-IDG/McGovern Institute for Brain Research, Beijing, China; 8 Shanghai Center for Brain Science and Brain-Inspired Intelligence Technology, Shangha, China

**Keywords:** Cell biology, Cell signalling

## Abstract

Astrocytes play a crucial role in regulating sleep–wake behavior, and adenosine signaling is generally thought to be involved. Here we show multiple lines of evidence supporting that modulation of the sleep–wake behavior by astrocyte Ca^2+^ activity could occur without adenosine signaling. In the basal forebrain and the brainstem, two brain regions that are known to be essential for sleep–wake regulation, chemogenetically-induced astrocyte Ca^2+^ elevation significantly modulated the sleep–wake cycle. Although astrocyte Ca^2+^ level positively correlated with the amount of extracellular adenosine, as revealed by a genetically encoded adenosine sensor, we found no detectable change in adenosine level after suppressing astrocyte Ca^2+^ elevation, and transgenic mice lacking one of the major extracellular ATP-adenosine conversion enzymes showed similar extracellular adenosine level and astrocyte Ca^2+^-induced sleep modulation. Furthermore, astrocyte Ca^2+^ is dependent primarily on local neuronal activity, causing brain region-specific regulation of the sleep–wake cycle. Thus, neural activity-dependent astrocyte activity could regulate the sleep–wake behavior independent of adenosine signaling.

## Introduction

Astrocytes are one of the most abundant cell types in the brain^1^. They provide essential support to the neural network and actively participate in many brain functions, including sleep–wake regulation^2–6^. Astrocytes have been proposed to modulate the sleep–wake cycle through multiple mechanisms^7^, among which adenosine signaling is generally believed to be critical^3,8^. Astrocyte-derived adenosine, produced by the hydrolysis of astrocyte-released ATP, was shown to regulate slow cortical oscillations^9^ and control the accumulation of sleep pressure^8,10^. In these studies, astrocyte-specific exocytosis was inhibited by expressing the dominant-negative domain of vesicular SNARE (dnSNARE) that was driven by the glial-fibrillary acidic protein (GFAP) promoter^11^.

In the dnSNARE mice, the involvement of astrocyte-derived adenosine in the sleep–wake regulation was indirectly validated via pharmacological blockade of adenosine receptors, and no direct measurement of extracellular adenosine level was performed, mainly due to the lack of sensitive tools to measure adenosine level in live animals. Additionally, the constitutive GFAP promoter used in these studies to achieve astrocyte-specific transgene expression has been shown to be not specific to astrocytes but also lead to considerable leaky expression in neurons^12–14^. These methodological concerns raise an important question of whether astrocyte-derived adenosine plays a critical role in sleep–wake regulation.

Extracellular adenosine can originate from ATP hydrolysis or equilibrative nucleoside transporter (ENT)-mediated release^15^. Both astrocytes and neurons have been implicated in these processes^15^, although their relative contribution is unclear. Our recent measurement of neuronal activity-dependent fast adenosine release in the basal forebrain (BF) using a novel GPCR-activation-based (GRAB) sensor for adenosine indicates that neurons represent an essential source of extracellular adenosine^16,17^, implicating that astrocyte-derived adenosine may constitute only a minor part of the extracellular adenosine. This also suggests that astrocyte-derived adenosine may not be critically involved in regulating sleep–wake behavior.

In the current study, we directly tested the involvement of adenosine signaling in sleep–wake regulation by astrocyte activity in two brain regions that are important for sleep–wake control. We examined the relationship between astrocyte Ca^2+^ transients and the dynamics of extracellular adenosine levels using fiber photometry recording and a newly developed adenosine sensor, and investigated the contributions of ATP hydrolysis-derived adenosine to the levels of extracellular adenosine and to the astrocyte Ca^2+^ elevation-induced sleep–wake modulation using transgenic mice lacking a critical enzyme that mediates ATP-adenosine conversion in the extracellular space. In addition, we examined the role of astrocyte activity in increasing extracellular adenosine levels and the control of the sleep–wake cycle, by directly suppressing the Ca^2+^ transients in BF astrocytes via conditional knockout of the receptors that are critical for mediating astrocyte Ca^2+^ activity. Our results strongly suggested that astrocytes in multiple brain regions can regulate the sleep–wake cycle independent of adenosine signaling pathways.

## Results

### Astrocyte-specific gene expression in the BF

**Fig. 1:**
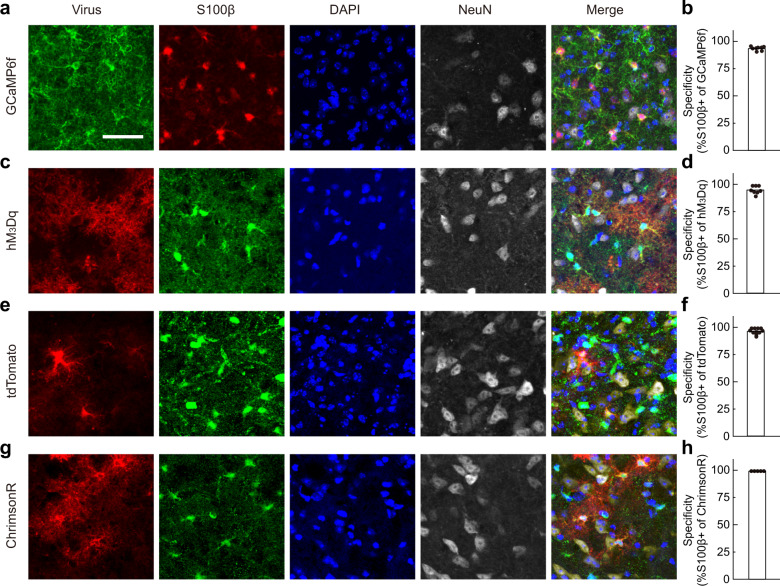
AAV5-GfaABC_1_D-mediated highly specific gene expression in BF astrocytes. **a** Immunohistochemical verification of GCaMP expression in BF astrocytes. **b** The histogram shows that 93.5% of the GCaMP positive astrocytes were S100β positive. *n* = 8 brain slices. Scale, 40 µm. **c**–**h** Same as **a** and **b**, respectively, except that hM_3_Dq, tdTomato, or ChrimsonR was expressed. The specificity for each group was 96.2%, 97.2%, or 100% and *n* = 8, 11, or 5 brain slices for each group, respectively.

Much controversy in the study of astrocyte function has arisen due to the non-specific leaky expressions of target genes into neurons, and the specificity of various mouse lines and virus-mediated methods differ in different brain regions^14^. Thus, we first tested several commonly used methods in order to achieve astrocyte-specific gene expression for recording and manipulating astrocyte activity in the BF. The BF was chosen because it is a well-documented brain region that plays a critical role in sleep–wake regulation^16,18,19^, and in which adenosine signaling is known to be important^16,20–22^. We found that the GFAP-Cre^12^, hGFAP-CreER^23^, and Aldh1l1-Cre^24^ mouse lines, that were reported to produce astrocyte-specific gene expression in many other brain regions^23–34^, all labeled cells with apparent neuron-like morphology in the BF (Supplementary Fig. S1), suggesting non-specific leaky expression in neurons. In contrast, a virus-mediated strategy (AAV5 + GfaABC_1_D promoter)^35–39^ constantly produced highly specific gene expression in astrocytes in the BF (Fig. 1). Immunohistochemical staining showed highly specific expression of various vectors that were used in the current study, with 93.5%–100% of the target gene overlapping with cells expressing the astrocyte marker S100β^35,36,40^ (Fig. 1).

Note that due to the notable heterogeneity of astrocytes in different brain regions, our finding that multiple approaches cause leaky expression of the transgene in neurons in the BF does not discredit previous studies using these approaches in other brain regions, as long as the specificity of transgene expression has been rigorously verified.

### Brain state-dependent Ca^2+^ activity in BF astrocytes

**Fig. 2:**
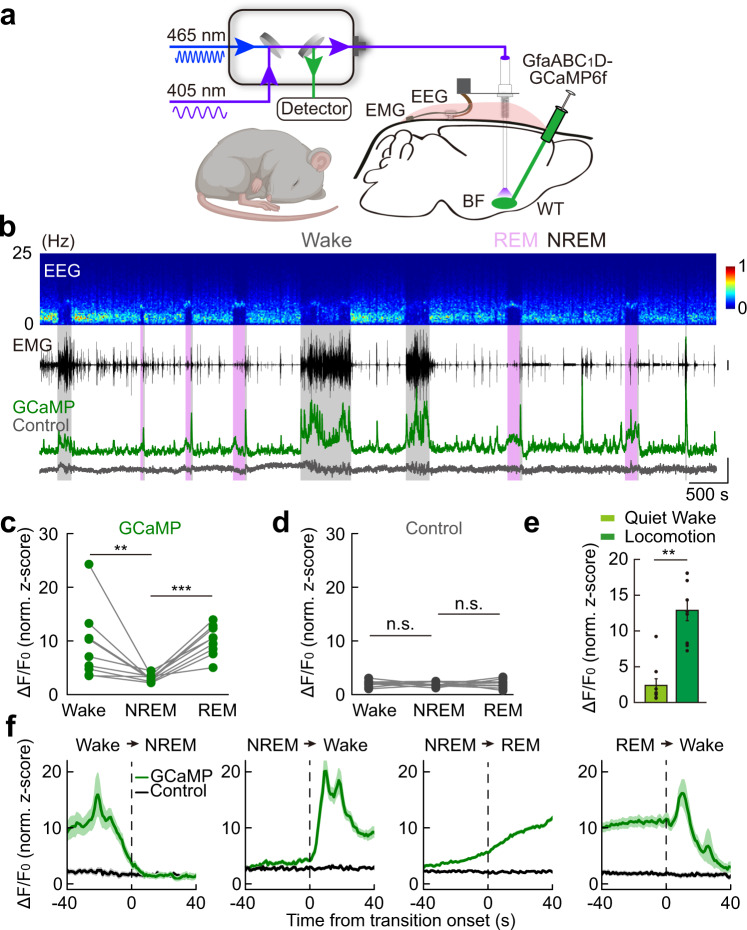
Brain state-dependent astrocyte Ca^2+^ activity in the BF during the sleep–wake cycle. **a** Schematic diagram depicting fiber photometry recording of astrocyte Ca^2+^ transients in the BF during the sleep–wake cycle. **b** Top to bottom: EEG power spectrogram; EMG (scale, 1 mV); photometry signals of GCaMP (green) and isosbestic excitation (gray) (scale, 1 *z*-score). The brain states are color-coded; the same color coding is used in all the following figures. **c** GCaMP fluorescence in different brain states. Each line represents data from one recording. *n* = 9 sessions from 4 mice; Wake vs NREM: **P = 0.0039 (Wilcoxon signed-rank test); REM vs NREM: ***P < 0.001 (Paired *t-*test). In this and all subsequent figures, summary data are expressed as the means ± SEM. **d** Fluorescence of the isosbestic excitation of GCaMP in different brain states. *n* = 9 sessions from 4 mice. n.s., not significant; Wake vs NREM: *P* = 0.74; REM vs NREM: *P* = 0.85; Paired *t-*test. **e** Astrocyte Ca^2+^ signal during quiet wakefulness and locomotion. *n* = 9 recording sections from 3 mice for both groups. **P = 0.0039 (Wilcoxon signed-rank test). **f** GCaMP and control signal during brain state transitions. The vertical dashed lines represent the transition time. *n* = 39, 134, 262, and 43 events from 4 mice for each panel, respectively.

Astrocyte activation is reflected by an elevation of intracellular Ca^2+^^41,42^. We thus first examined the dynamics of astrocyte Ca^2+^ transients during the sleep–wake cycle in the BF using in vivo fiber photometry recording of genetically encoded Ca^2+^ indicator, GCaMP6f^43^, while simultaneously recording EEG and EMG in free-moving mice (Fig. 2a). The GCaMP6f was expressed in astrocytes of the mouse BF via local injection of a viral vector expressing GfaABC_1_D-GCaMP6f^37,44^ (Supplementary Fig. S2).

The population Ca^2+^ activity in astrocytes showed dynamic changes in a brain state-dependent manner (Fig. 2b, c), which was absent in a control condition in which the GCaMP was illuminated with isosbestic excitation light (405 nm) (Fig. 2b, d)^45,46^. The astrocyte Ca^2+^ activity was high during both wakefulness and rapid-eye-movement (REM) sleep and low during non-REM (NREM) sleep (Fig. 2c; Wake vs NREM: **P = 0.0039, Wilcoxon signed-rank test; REM vs NREM: ***P < 0.001, Paired *t-*test), showing rapid dynamics during brain state transitions (Fig. 2f). There was also a high correlation between astrocyte Ca^2+^ activity and locomotion of the mice (Fig. 2e, **P = 0.0039, Wilcoxon signed-rank test)^47–52^. This vigilant state-dependent, rapid dynamic change of astrocyte Ca^2+^ activity during the sleep–wake cycle is highly similar to previously reported neuronal activity in BF^16,19^, suggesting that astrocyte and neuronal activity dynamics are closely correlated. Such dynamic change of astrocyte Ca^2+^ activity in the BF is also largely consistent with previous measurements in the cortex^51–53^, hippocampus^54^, and several subcortical regions^54^.

### Sleep–wake modulation by astrocyte Ca^2+^ activity in the BF

**Fig. 3:**
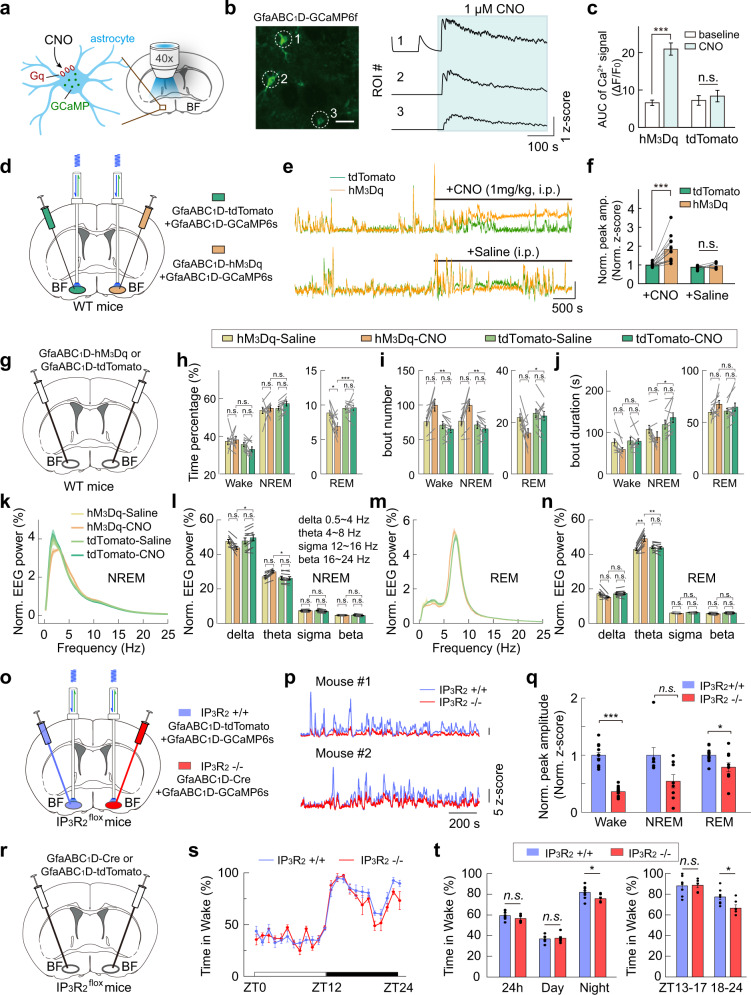
Sleep–wake modulation by astrocyte Ca^2+^ activity in the BF. **a** Schematic diagram depicting Ca^2+^ imaging from hM_3_Dq-expressing astrocytes in brain slices. **b** Left, confocal image showing GCaMP-expressing astrocyte in a brain slice (scale, 50 µm); right, Ca^2+^ signals of the individual region of interest (ROI). **c** Quantification of CNO-evoked Ca^2+^ signals. AUC, area under the curve. AUC measures the area of Ca^2+^ signals during baseline (the 2-min period prior to CNO infusion) and after bath application of CNO (2 min). hM_3_Dq group: *n* = 99 ROIs; ***P < 0.001. tdTomato group: *n* = 51 ROIs; *P* = 0.093. (Paired *t-*test). **d**–**f** Chemogenetic activation of BF astrocytes in live mice. **d** Schematic diagram depicting fiber photometry recording of chemogenetic activation-induced astrocyte Ca^2+^ signal. AAVs expressing GfaABC_1_D-tdTomato and GfaABC_1_D-GCaMP6s were injected into the BF of one hemisphere, and AAVs expressing GfaABC_1_D-hM3Dq and GfaABC_1_D-GCaMP6s were injected into the contralateral BF. **e** Example traces showing astrocyte Ca^2+^ signal from the BF expressing tdTomato (green) or hM_3_Dq (yellow), respectively. Free-behaving mice were injected with CNO (1 mg/kg, i.p.) or saline (i.p.). Scale, 2 normalized *z*-score. **f** Quantitative comparison of astrocyte Ca^2+^ signal from the BF expressing tdTomato (green) or hM_3_Dq (yellow) in response to CNO or saline injections. The mean ratio between the size of astrocyte Ca^2+^ events from the hM_3_Dq- and tdTomato-expressing BF was calculated before and after CNO or saline injections. CNO injection significantly increased the size of GCaMP events in hM_3_Dq-expressing BF (186% increase, ***P < 0.001, Paired *t-*test), whereas saline injection had no detectable effect (*P* = 0.43, Paired *t-*test). CNO injections, *n* = 13 sessions from 4 mice; Saline injections, *n* = 7 sessions from 4 mice. **g** Schematic diagram depicting chemogenetic activation of astrocytes in the BF in wide-type (WT) mice. **h**–**n** Chemogenetic activation of BF astrocytes in WT mice significantly changed sleep–wake behavior (within the 4 h after CNO injection). hM_3_Dq group, *n* = 9 mice; tdTomato group, *n* = 10 mice. The statistical method was two-way repeated measures ANOVA, followed by Tukey’s post hoc multiple comparison test. Time percentage (**h**), bout number (**i**), and bout duration (**j**) of the three brain states after CNO or saline injections. In **h**, hM_3_Dq-CNO vs tdTomato-CNO: Wake, *P* = 0.084; NREM, *P* = 0.57; REM, ***P < 0.001. hM_3_Dq-Saline vs hM_3_Dq-CNO: Wake, *P* = 0.98; NREM, *P* = 0.92; REM, *P = 0.012. tdTomato-Saline vs tdTomato-CNO: Wake, *P* = 0.54; NREM, *P* = 0.49; REM, *P* = 1.0. In **i**, hM_3_Dq-CNO vs tdTomato-CNO: Wake, **P = 0.0026; NREM, **P = 0.0028; REM, *P = 0.033. hM_3_Dq-Saline vs hM_3_Dq-CNO: Wake, *P* = 0.051; NREM, *P* = 0.054; REM, *P* = 0.065. tdTomato-Saline vs tdTomato-CNO: Wake, *P* = 0.87; NREM, *P* = 0.88; REM, *P* = 0.96. In **j**, hM_3_Dq-CNO vs tdTomato-CNO: Wake, *P* = 0.22; NREM, *P = 0.029; REM, *P* = 0.91. hM_3_Dq-Saline vs hM_3_Dq-CNO: Wake, *P* = 0.34; NREM, *P* = 0.62; REM, *P* = 0.31. tdTomato-Saline vs tdTomato-CNO: Wake, *P* = 1.0; NREM, *P* = 0.67; REM, *P* = 0.79. **k** Normalized NREM EEG power after the injection of CNO or saline in hM3Dq- or tdTomato-expressing mice. **l** Quantification of NREM EEG power in delta, theta, sigma, and beta bands. hM_3_Dq-CNO vs tdTomato-CNO: delta, *P = 0.039; theta, *P = 0.014; sigma, *P* = 0.54; beta, *P* = 0.60. hM_3_Dq-Saline vs hM_3_Dq-CNO: delta, *P* = 0.27; theta, *P* = 0.083; sigma, *P* = 1.0; beta, *P* = 1.0. tdTomato-Saline vs tdTomato-CNO: delta, *P* = 0.79; theta, *P* = 0.99; sigma, *P* = 0.46; beta, *P* = 0.42. **m**, **n** Same as **k**, **l**, except that REM EEG was analyzed. In **n**, hM_3_Dq-CNO vs tdTomato-CNO: delta, *P* = 0.065; theta, **P = 0.0041; sigma, *P* = 0.16; beta, *P* = 0.17. hM_3_Dq-Saline vs hM_3_Dq-CNO: delta, *P* = 0.091; theta, **P = 0.0012; sigma, *P* = 0.71; beta, *P* = 0.86. tdTomato-Saline vs tdTomato-CNO: delta, *P* = 0.99; theta, *P* = 1.0; sigma, *P* = 1.0; beta, *P* = 1.0. **o**–**q** IP_3_R_2_ knockout (KO) reduced astrocyte Ca^2+^ activity during both wakefulness and REM sleep. **o** Schematic diagram depicting fiber photometry recording of astrocyte Ca^2+^ signal in the BF to examine the effect of IP_3_R_2_-KO. **p** Example astrocyte Ca^2+^ signal from the BF expressing Cre (red, IP_3_R_2_^−/−^) or control virus (light blue, IP_3_R_2_^+/+^), respectively. Scale bar = 200 s and 5 *z*-score. **q** Quantitative comparison of astrocyte Ca^2+^ signal from the BF expressing Cre (red, IP_3_R_2_^−/−^) or control virus (light blue, IP_3_R_2_^+/+^) during different brain states. Wake, reduced by 64%, ***P < 0.001, Paired *t-*test; NREM, *P* = 0.078, Wilcoxon signed-rank test; REM, reduced by 21%, *P = 0.021, Paired *t-*test; *n* = 12 recordings from 4 mice (3 recordings per mouse). **r**–**t** IP_3_R_2_-KO in BF astrocytes reduced wakefulness in the late phase of the night. **r** Schematic diagram of suppressing astrocyte Ca^2+^ elevation in the BF using the IP_3_R_2_^*flox*^ mice. **s** Circadian variation of wakefulness in the two groups. *n* = 6 and 8 mice for Cre group and tdTomato group, respectively. **t** Percentage of wakefulness in the entire 24 h, during the day, during the night, and the early (ZT13–17) or late phase (ZT18–24) of the night. 24 h, *P* = 0.21; Day, *P* = 0.79; Night, *P = 0.049; ZT13–17, *P* = 0.90; ZT18–24, *P = 0.022; Student’s *t-*test.

We next examined how the Ca^2+^ elevation in BF astrocytes contributes to sleep–wake regulation by elevating astrocyte Ca^2+^ with a chemogenetic method^55^. We first injected a viral vector that specifically expressed GfaABC_1_D-hM_3_Dq (a designer receptor exclusively activated by designer drugs, DREADDs)^55,56^ in BF astrocytes (Fig. 3a–n and Supplementary Fig. S3), and 2 weeks later, we injected the hM_3_Dq ligand, clozapine-*N*-oxide (CNO), intraperitoneally (i.p.) in the mice to evoke Ca^2+^ transients in the astrocytes. Mice injected with virus expressing GfaABC_1_D-tdTomato were used as the control group. We confirmed that CNO application produced a large elevation of both tonic and phasic Ca^2+^ activity in astrocytes using both brain slices (Fig. 3a–c) and live mouse preparations (Fig. 3d–f).

To quantify sleep–wake regulation by chemogenetically-induced astrocyte Ca^2+^ activation, we calculated statistical significance using the two-way repeated measures ANOVA with group (hM_3_Dq vs tdTomato) and treatment (CNO vs saline) as factors, followed by Tukey’s post hoc multiple comparison test when significant main effects were found (*α* = 0.05). The statistical approach was chosen because CNO has been shown to be metabolized into clozapine^57,58^, which activates multiple monoamine receptors implicated in sleep–wake regulation; two-way ANOVA can unmask the nonspecific effects of CNO and reveal the effects of astrocyte Ca^2+^ activation. In our experiments, we consistently observed modest increases in NREM sleep following injection of low doses of CNO (0.5–1 mg/kg) in tdTomato-expressing mice. Therefore, in all the following behavioral experiments involving CNO treatment, we mainly report the statistical results from Tukey’s post hoc test between the hM_3_Dq-CNO group and tdTomato-CNO, unless otherwise stated.

The chemogenetically-induced astrocyte Ca^2+^ elevation significantly changed the sleep–wake behavior of the mice (Fig. 3g–j and Supplementary Fig. S3c). The most prominent effect was a decrease in the total time of REM sleep in the hM_3_Dq-CNO group (Fig. 3h; decreased by 28%; ***P < 0.001), without apparent changes in the duration of wakefulness or NREM sleep (Fig. 3h; Wake, *P* = 0.084; NREM, *P* = 0.57; two-way repeated measures ANOVA). The reduction in REM sleep was not observed in mice injected with the control virus expressing GfaABC_1_D-tdTomato (Fig. 3h; *P* = 1.0). Further analysis suggested that the reduction in REM sleep was mainly due to the reduction of REM bouts (Fig. 3i; *P = 0.033), which was likely caused by increased fragmentation of the NREM sleep, as shown by the increased bout number of NREM and wakefulness and decreased bout duration of NREM after CNO injection (Fig. 3i, j; bout number: NREM, **P = 0.0028; Wake, **P = 0.0026; bout duration: NREM, *P = 0.029; Wake, *P* = 0.22).

The EEG spectrum during sleep was also changed by chemogenetic activation of the BF astrocytes (Fig. 3k–n). There were significant decrease of NREM delta power (0.5–4 Hz) (Fig. 3l; *P = 0.039) and increase of theta power (4–8 Hz) during both NREM and REM sleep (Fig. 3l, n; NREM, *P = 0.014; REM, **P = 0.0041). These results suggest that astrocyte activation in the BF can modulate the sleep–wake cycle by decreasing the consolidation and depth of NREM sleep, as indicated by increased NREM fragmentation and decreased NREM delta power, respectively.

To further address the physiological role of the BF astrocytes in sleep–wake regulation, we performed a loss-of-function experiment (Fig. 3o–t and Supplementary Fig. S4) by selectively suppressing Ca^2+^ elevation in BF astrocytes using the IP_3_R_2_^flox^ mice^59^, which provides genetic access to the receptor, inositol 1,4,5-trisphosphate (IP_3_) receptor type 2 (IP_3_R_2_), that mediates a significant part of Ca^2+^ increase in astrocyte^60^. Injection of GfaABC_1_D-Cre into the BF of the IP_3_R_2_^flox^ mice suppressed astrocyte Ca^2+^ transient during the sleep–wake cycle, with a marked decrease in wakefulness (64% reduction, ***P < 0.001, Paired *t-*test), moderate decrease in REM sleep (21% reduction, *P = 0.021, Paired *t-*test), and no significant change in NREM sleep (*P* = 0.078, Wilcoxon signed-rank test) (Fig. 3p, q). Bilateral injection of GfaABC_1_D-Cre in the BF of the IP_3_R_2_^flox^ mice (Fig. 3r and Supplementary Fig. S4a, b) caused a significant decrease in wakefulness, especially in the late phase of the night (Fig. 3s, t; ZT18−24 *P = 0.022, Student’s *t-*test), without affecting the sleep–wake states during other time of the day (Fig. 3s, t and Supplementary Fig. S4c−f). These results indicated that BF astrocytes play a role in supporting wakefulness under physiological conditions.

### Astrocyte Ca^2+^ activity in the BF is highly correlated with the extracellular levels of ATP and adenosine

We next tested the involvement of adenosine signaling in the astrocyte Ca^2+^ elevation-induced sleep–wake modulation. To measure extracellular adenosine levels, we used a genetically encoded sensor, GRAB_Ado_^16^. GRAB_Ado_ is an engineered chimera protein that is made by replacing the 3rd intracellular loop of the adenosine A_2A_ receptor with a conformation-sensitive circularly permuted enhanced GFP (cpEGFP), so that the amount of extracellular adenosine is indicated by the cpGFP fluorescence intensity. GRAB_Ado_ inherits the molecule selectivity of the adenosine A_2A_ receptor and has rapid response kinetics, with a rising time constant of 70 ms and high sensitivity of 60 nM as measured by the median effective concentration (EC_50_). Its fluorescence intensity correlates with adenosine level within a broad range (~1 nM–10 µM), which covers the extracellular adenosine level under physiological conditions (estimated between ten and a few hundred nM)^61,62^. The measurement of extracellular ATP level was achieved by using the ATP GRAB sensor (GRAB_ATP_)^63^.

**Fig. 4:**
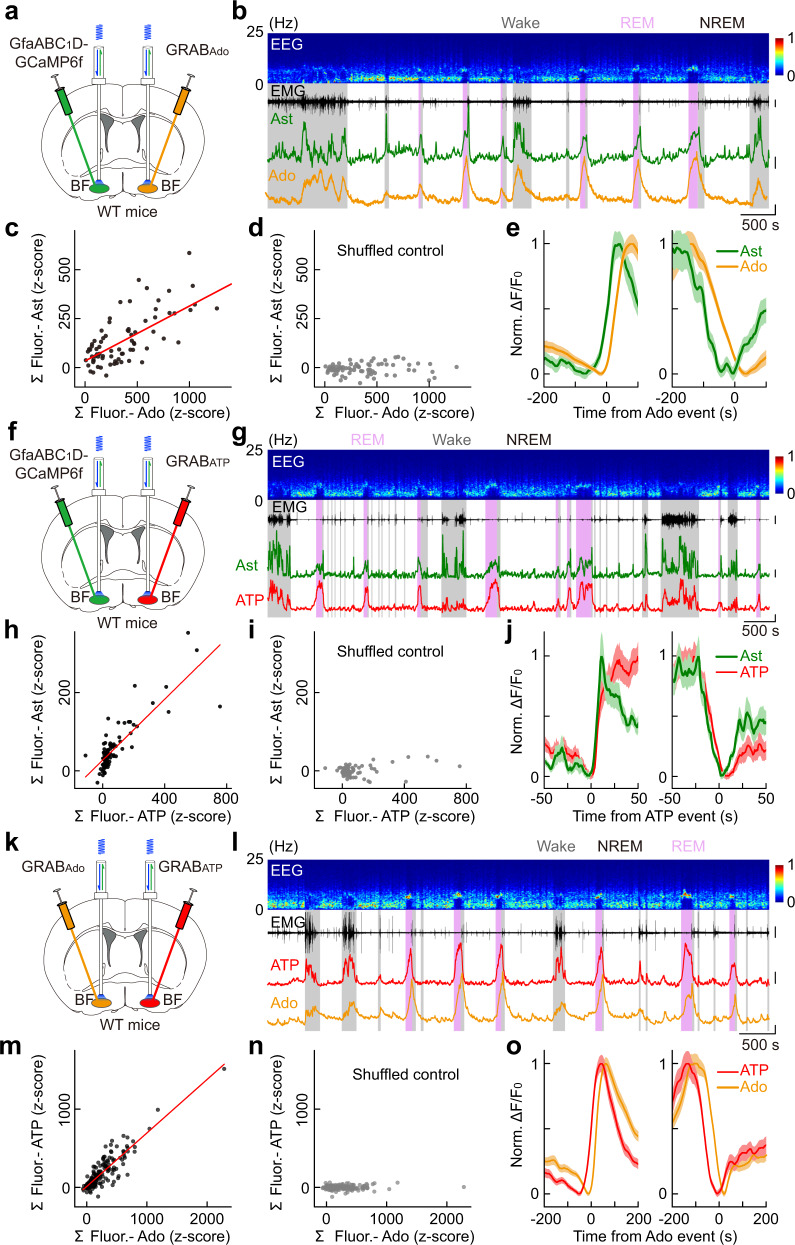
Astrocyte Ca^2+^ elevation in the BF is highly correlated with the dynamics of extracellular adenosine and ATP levels. **a** Schematic diagram depicting fiber photometry recording of extracellular adenosine level and population Ca^2+^ signal of astrocytes in the BF during the sleep–wake cycle. **b** Top to bottom, EEG power spectrogram, EMG (scale, 0.5 mV), GCaMP fluorescence (scale, 1 *z*-score), and GRAB_Ado_ fluorescence (scale, 1 *z*-score). **c** Correlation between the size of GCaMP and GRAB_Ado_ events. The red line represents a linear fit. *n* = 76 events from 7 recordings in 4 mice. Pearson’s *r* = 0.69, ***P < 0.001. **d** Same as in **c** after the GCaMP signal was randomly shuffled. Pearson’s *r* = 0.07. **e** Time course of the GCaMP and GRAB_Ado_ signal aligned to the onset (left) or offset (right) of the GRAB_Ado_ events. **f**–**j** Same as **a**–**e**, respectively, except that population Ca^2+^ signal of astrocytes and extracellular ATP were measured. Scale bar in **g**: EMG, 2 mV; GCaMP, 1 *z*-score; GRAB_ATP_, 1 *z*-score. In **h**: *n* = 88 events from 8 recordings in 4 mice. Pearson’s *r* = 0.84, ***P < 0.001. In **I**: Pearson’s *r* = 0.32. **k**–**o** Same as **a**–**e**, respectively, except that extracellular adenosine and ATP were measured. Scale bar in **l**: EMG, 0.5 mV; GRAB_Ado_, 1 *z*-score; GRAB_ATP_, 1 *z*-score. In **m**: *n* = 199 events from 15 recordings in 4 mice. Pearson’s *r* = 0.89, ***P < 0.001. In **n**: Pearson’s *r* = 0.10.

Whether the astrocyte Ca^2+^ transients correlate with the change in extracellular adenosine was examined by fiber photometry recording with the “bilateral dual probes” method^16^ that simultaneously monitored astrocyte Ca^2+^ (with GCaMP6f) and extracellular adenosine (with GRAB_Ado_) in the BF of two different hemispheres (Fig. 4a and Supplementary Fig. S5a, b). AAVs expressing GfaABC_1_D-GCaMP6f were injected into one hemisphere of the BF, and AAVs expressing hSyn-GRAB_Ado_ were injected into the contralateral hemisphere of the BF. This dual-probe method allows the simultaneous recording of fluorescence signals with an overlapping emission wavelength in the same brain region. The GRAB_Ado_ sensor has widely distributed expression, so it can report adenosine release in both soma and processes of neurons or astrocytes^16,64^. We found that, during the sleep–wake cycle, the spontaneous increase of astrocyte Ca^2+^ activity and extracellular adenosine had a similar time course (Fig. 4b), with the magnitude of the two signals highly correlated (Fig. 4c; Pearson’s *r* = 0.69, ***P < 0.001). Such correlation was not observed in the control analysis, in which the GCaMP signals were temporally shuffled in a random manner (Fig. 4d; Pearson’s *r* = 0.07) ^16^. More importantly, changes in astrocyte Ca^2+^ activity preceded that of the adenosine signal by approximately 23 s (Fig. 4e), suggesting that astrocyte activity may regulate the amount of extracellular adenosine^8,11^.

Astrocyte-derived adenosine is known to be produced by hydrolysis of astrocyte-released ATP in the extracellular space^11,65,66^. We thus next measured the dynamics of astrocyte Ca^2+^ activity and extracellular ATP level simultaneously in the BF, using the same “bilateral dual probes” method (Fig. 4f and Supplementary Fig. S5c, d). We found that the astrocyte Ca^2+^ and ATP signals were highly correlated in the time course and the magnitude (Fig. 4g−j). Interestingly, the change in ATP signal immediately followed the change in astrocyte Ca^2+^ signal with a delay of about 1 s (Fig. 4j). This result is consistent with previous findings that astrocytes are an important source of extracellular ATP^15^.

The measurements of astrocyte Ca^2+^ activity and extracellular adenosine and ATP levels predict that changes in ATP levels may precede changes in adenosine levels by approximately 22 s. Consistent with this prediction, our simultaneous recording of extracellular adenosine and ATP levels in the BF using the “bilateral dual probes” method revealed an actual difference of approximately 22 s (Fig. 4k−o and Supplementary Fig. S5e, f), furthering supporting that ATP release may underlie the increase of extracellular adenosine^15^.

### Optogenetically evoked astrocyte Ca^2+^ elevation causes no detectable adenosine increase

To directly examine the causal relationship between astrocyte Ca^2+^ elevation and extracellular adenosine increase in the BF, we evoked astrocyte Ca^2+^ activity optogenetically and used fiber photometry to measure the adenosine level by local injection of AAVs expressing GRAB_Ado_ and astrocyte-specific red-shifted channelrhodopsin, GfaABC_1_D-ChrimsonR^67^ (Supplementary Fig. S6). Although laser light (638 nm laser, 10 ms/pulse, 10 Hz for 10 s) could reliably induce a significant increase in astrocyte Ca^2+^ elevation (Supplementary Fig. S7; **P = 0.0011, Student’s *t-*test), we found no detectable increase in extracellular adenosine after laser application (Supplementary Fig. S6d; *P* = 0.39, Student’s *t-*test), in contrast to the previously reported prominent adenosine increase by optogenetic neuronal activation in the same brain region^16^. Thus, astrocyte Ca^2+^ elevation does not always induce an increase in extracellular adenosine. This result raises the possibility that astrocyte Ca^2+^ activity may not be the major cause of the observed extracellular adenosine increase in the BF during the sleep–wake cycle.

The use of optogenetic methods provided a time-locked stimulation-analysis window, thus ensuring highly sensitive measurements of the causal relationship between astrocyte Ca^2+^ elevation and extracellular adenosine increase. However, it is also possible that optogenetic stimulation-induced astrocyte Ca^2+^ differs from that occurs physiologically during the sleep–wake cycle, as reported previously^68,69^. We thus performed additional experiments to further examine the involvement of astrocyte-derived adenosine in the modulation of the sleep–wake cycle.

### Adenosine level dynamics and astrocyte Ca^2+^ elevation-induced sleep–wake modulation are not affected in the CD73-KO mice

**Fig. 5:**
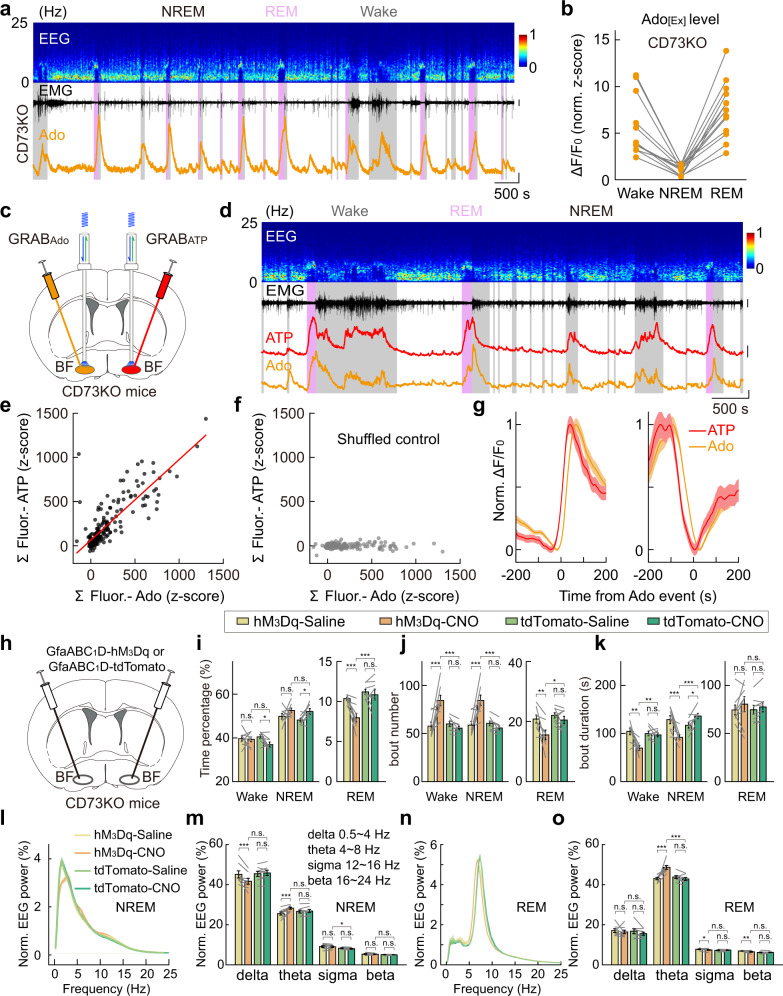
CD73-KO mice show normal extracellular adenosine dynamics and astrocyte Ca^2+^ elevation-induced modulation to the sleep–wake cycle. **a** Recording of adenosine signal in the BF during the sleep–wake cycle in CD73-KO mice. Top to bottom: EEG power spectrogram, EMG (scale, 1 mV), and GRAB_Ado_ fluorescence (scale, 1 *z*-score). **b** GRAB_Ado_ signal in CD73-KO mice in different brain states. Each line represents data from one recording. *n* = 13 sessions from 6 mice. CD73KO vs WT (reported previously in Fig. 4l in Peng et al.): Wake, *P* = 0.41; REM, *P* = 0.76; Wilcoxon rank-sum test. **c–g** Same as Fig. 4**k**–**o**, respectively, except that experiments were performed using CD73-KO mice. Scale bar in **d**: EMG, 1 mV; GRAB_Ado_, 1 *z*-score; GRAB_ATP_, 1 *z*-score. In **e**: *n* = 187 events from 14 recordings in 4 mice. Pearson’s *r* = 0.83, ***P < 0.001. In **f**: Pearson’s *r* = 0.07. **h**–**o** Same as Fig. 3**g**–**n**, respectively, except that experiments were performed using CD73-KO mice. hM_3_Dq group, *n* = 8 mice; tdTomato group, *n* = 8 mice. The statistical method was two-way repeated measures ANOVA, followed by Tukey’s post hoc multiple comparison test. In **i**, hM_3_Dq-CNO vs tdTomato-CNO: Wake, *P* = 0.14; NREM, *P* = 0.78; REM, ***P < 0.001. hM_3_Dq-Saline vs hM_3_Dq-CNO: Wake, *P* = 0.85; NREM, *P* = 0.15; REM, ***P < 0.001. tdTomato-Saline vs tdTomato-CNO: Wake, *P = 0.035; NREM, *P = 0.042; REM, *P* = 0.51. In **j**, hM_3_Dq-CNO vs tdTomato-CNO: Wake, ***P < 0.001; NREM, ***P < 0.001; REM, *P = 0.017. hM_3_Dq-Saline vs hM_3_Dq-CNO: Wake, ***P < 0.001; NREM, ***P < 0.001; REM, **P = 0.0020. tdTomato-Saline vs tdTomato-CNO: Wake, *P* = 0.42; NREM, *P* = 0.38; REM, *P* = 0.30. In **k**, hM_3_Dq-CNO vs tdTomato-CNO: Wake, **P = 0.0028; NREM, ***P < 0.001; REM, *P* = 0.73. hM_3_Dq-Saline vs hM_3_Dq-CNO: Wake, **P = 0.0011; NREM, ***P < 0.001; REM, *P* = 0.21. tdTomato-Saline vs tdTomato-CNO: Wake, *P* = 0.75; NREM, *P = 0.022; REM, *P* = 0.52. In **m**, hM_3_Dq-CNO vs tdTomato-CNO: delta, *P* = 0.063; theta, *P* = 0.18; sigma, *P = 0.045; beta, *P* = 0.18. hM_3_Dq-Saline vs hM_3_Dq-CNO: delta, ***P < 0.001; theta, ***P < 0.001; sigma, *P* = 0.50; beta, *P* = 0.58. tdTomato-Saline vs tdTomato-CNO: delta, *P* = 0.54; theta, *P* = 0.62; sigma, *P* = 0.064; beta, *P* = 0.52. In **o**, hM_3_Dq-CNO vs tdTomato-CNO: delta, *P* = 0.53; theta, ***P < 0.001; sigma, *P* = 0.89; beta, *P* = 0.36. hM_3_Dq-Saline vs hM_3_Dq-CNO: delta, *P* = 0.47; theta, ***P < 0.001; sigma, *P = 0.023; beta, **P = 0.0076. tdTomato-Saline vs tdTomato-CNO: delta, *P* = 0.20; theta, *P* = 0.35; sigma, *P* = 0.28; beta, *P* = 0.69.

Astrocytes are thought to increase extracellular adenosine by releasing ATP, which is then hydrolyzed to adenosine by a series of ecto-enzymes^70,71^, including the key enzyme CD73 (encoded by *Nt5e*) that converts AMP to adenosine^17,72^. If astrocyte-derived ATP represents a significant contributor to extracellular adenosine during the sleep–wake cycle, we would expect that blocking CD73 activity would substantially reduce the amount of extracellular adenosine and its correlation with extracellular ATP. However, in the BF of CD73 KO mice^73,74^, using fiber photometry recording of GRAB_Ado_, we detected a notable increase in adenosine signal during both wakefulness and REM sleep, with no significant difference from that previously reported in the WT mice^16^ (Fig. 5a, b and Supplementary Fig. S8a, b; CD73-KO vs WT: Wake, *P* = 0.41; REM, *P* = 0.76; Wilcoxon rank-sum test; Data for WT mice were reported previously in Fig. 4l of Peng et al.^16^). This result indicates that CD73-mediated, ATP hydrolysis-derived adenosine does not contribute significantly to the amount of extracellular adenosine in the BF during the sleep–wake cycle^17,75,76^.

Further support for this conclusion was provided by our finding that bilateral dual-probe measurements of extracellular adenosine and ATP in the BF of CD73-KO mice during the sleep–wake cycle (Fig. 5c–g and Supplementary Fig. S8c, d) showed that the dynamics of extracellular ATP and adenosine was not significantly different from that of the WT mice, with the two signals remained highly correlated in both the time course and magnitude.

In line with these results, the hM_3_Dq-induced astrocyte Ca^2+^ elevation in the BF caused comparable modulation to the sleep–wake behavior in CD73-KO mice as that in WT mice (Fig. 5h–k and Supplementary Fig. S9), with a significant decrease in the total time of REM sleep (Fig. 5i; reduced by 26%, ***P < 0.001), REM bout number (Fig. 5j; *P = 0.017), and fragmented NREM sleep as indicated by the increased bout number and decreased bout duration of both NREM and wakefulness (Fig. 5j, k; bout number: NREM, ***P < 0.001; Wake, ***P < 0.001; bout duration: NREM, ***P < 0.001; Wake, **P = 0.0028). Moreover, the EEG spectrum also exhibited similar changes as that in WT mice (Fig. 5l–o) (EEG delta power: NREM, *P* = 0.063; REM, *P* = 0.53; EEG theta power: NREM, *P* = 0.18; REM, ***P < 0.001).

### Suppressing Ca^2+^ elevation in BF astrocytes decreases the extracellular levels of ATP but not adenosine

**Fig. 6:**
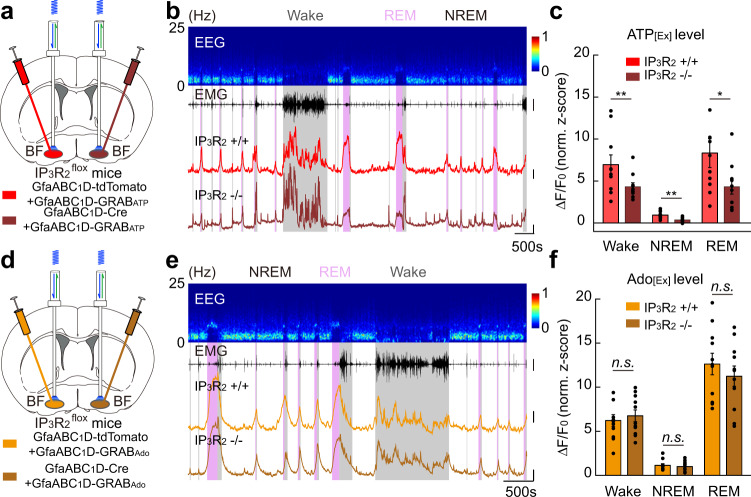
Suppressing Ca^2+^ elevation in BF astrocytes decreases extracellular level of ATP but not adenosine. **a** Schematic diagram depicting fiber photometry recording of extracellular ATP level in the BF of IP_3_R_2_^flox^ mice during the sleep–wake cycle. IP_3_R_2_ receptors from astrocytes were knocked out in one hemisphere of the BF via injection of GfaABC_1_D-Cre, and extracellular ATP level from the two hemispheres of the same mouse was compared. **b** Top to bottom, EEG power spectrogram, EMG (scale, 2 mV), and GRAB_ATP_ fluorescence of the two hemispheres, respectively (scale, 1 *z*-score and 500 s). **c** Quantification of GRAB_ATP_ fluorescence in the two hemispheres. *n* = 10 sessions from 4 mice. Wake, **P = 0.0098, Wilcoxon signed-rank test; NREM, **P = 0.0033, Paired *t-*test; REM, *P = 0.042, Paired *t-*test. **d**–**f** Same as **a**–**c**, respectively, except that extracellular adenosine level was measured. Scale bar in **e**: EMG, 2 mV; GRAB_Ado_, 1 *z*-score. In **f**: *n* = 11 sessions from 4 mice. Wake, *P* = 0.48, Paired *t-*test; NREM, *P* = 0.27, Wilcoxon signed-rank test; REM, *P* = 0.34, Paired *t-*test.

Because an alternative explanation for our above observations could be that extracellular ATP–adenosine conversion in the BF is mediated by other ectoenzymes rather than CD73, we directly tested the contribution of BF astrocytes to the elevation of extracellular adenosine by suppressing the astrocyte Ca^2+^ elevation using the IP_3_R_2_^flox^ mice^59^. We selectively deleted the IP_3_R_2_ receptors from BF astrocytes by injecting a viral vector expressing GfaABC_1_D-Cre and compared the extracellular level of ATP and adenosine with that in the contralateral BF (injected with a control vector expressing GfaABC_1_D-tdTomato) in the same mouse (Fig. 6 and Supplementary Fig. S10). This IP_3_R_2_-KO-induced suppression of astrocyte Ca^2+^ elevation caused a significant decrease of the extracellular level of ATP (Fig. 6b, c) (Wake, **P = 0.0098, Wilcoxon signed-rank test; NREM, **P = 0.0033, Paired *t-*test; REM, *P = 0.042, Paired *t-*test), consistent with the notion that astrocytes are an important source of extracellular ATP^65,77^; however, we observed no detectable change in the extracellular level of adenosine (Fig. 6e, f) (Wake, *P* = 0.48, Paired *t-*test; NREM, *P* = 0.27, Wilcoxon signed-rank test; REM, *P* = 0.34, Paired *t-*test). This uncorrelated change in the amount of extracellular ATP and adenosine further supported the above notion that ATP hydrolysis-derived adenosine does not contribute significantly to the amount of extracellular adenosine in the BF during the sleep–wake cycle. These results strongly suggested that astrocyte activity is not a significant factor in increasing extracellular adenosine in the BF during the sleep–wake cycle, further implying that astrocyte Ca^2+^ elevation may modulate the sleep–wake behavior through adenosine-independent pathways.

### Ca^2+^ elevation in BF astrocyte modulates local neural network

**Fig. 7:**
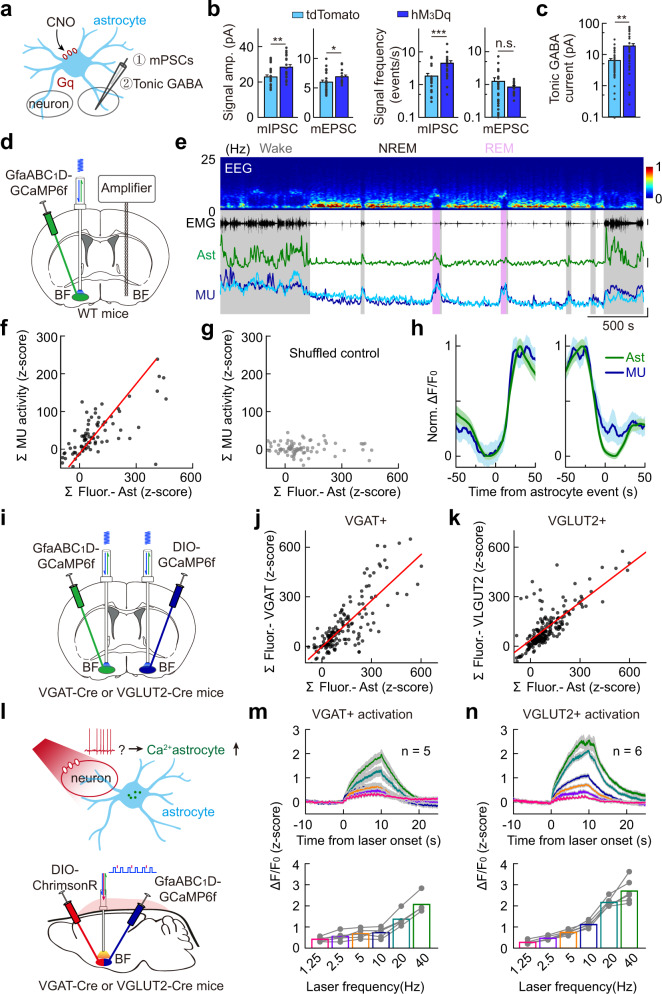
Astrocyte–neuron interactions in the BF. **a** Schematic diagram depicting patch-clamp recording of BF neurons after activation of hM_3_Dq-expressing astrocytes. **b** CNO applications altered miniature synaptic transmissions in hM_3_Dq-expressing brain slices. mIPSC: Amplitude, **P = 0.005, Student’s *t-*test; Frequency, ***P < 0.001, Wilcoxon rank-sum test; *n* = 22 and 23 cells for hM_3_Dq and tdTomato group, respectively. mEPSC: Amplitude, *P = 0.041, Student’s *t-*test; Frequency, *P* = 0.48, Wilcoxon rank-sum test. *n* = 20 and 24 cells for hM_3_Dq and tdTomato group, respectively. **c** CNO applications increased tonic GABA current in hM_3_Dq-expressing brain slice. **P = 0.0011, Wilcoxon rank-sum test; *n* = 38 and 37 cells for hM_3_Dq and tdTomato group, respectively. **d** Schematic diagram depicting simultaneous recording of population Ca^2+^ signal of astrocytes and multi-unit neural activity in the BF during the sleep–wake cycle. **e** Top to bottom, EEG power spectrogram, EMG (scale, 0.1 mV), GCaMP fluorescence (scale, 1 *z*-score), and multi-unit neural activity (scale, 1 *z*-score). **f**–**h** Same as Fig. 4**c**–**e**, respectively, except that extracellular Ado and multi-unit neural activity were compared. *n* = 80 events from 9 recordings in 3 mice. In **f**, Pearson’s *r* = 0.70, ***P < 0.001; in **g**, Pearson’s *r* = –0.01. **i** Schematic diagram depicting simultaneous recording of population Ca^2+^ signal of astrocytes and VGAT^+^ or VGLUT2^+^ neurons in the BF during the sleep–wake cycle. **j** Correlation between the size of GCaMP events in astrocytes and VGAT^+^ neurons. The red line represents a linear fit. *n* = 156 events from 8 recordings in 3 mice. Pearson’s *r* = 0.80, ***P < 0.001. **k** Same as **j**, except that GCaMP events in astrocytes and VGLUT2^+^ neurons were compared. *n* = 139 events from 9 recordings in 3 mice. Pearson’s *r* = 0.76, ***P < 0.001. **l** Schematic diagram depicting fiber photometry recording of GCaMP signals in astrocytes induced by optogenetic activation of VGAT^+^ or VGLUT2^+^ neurons. **m** Summary of evoked GCaMP signals in astrocytes induced by optogenetic activation of VGAT^+^ neurons using different frequencies (shown in different colors) of laser pulses. *n* = 5 mice. **n** Same as **m**, except that VGLUT2^+^ neurons were activated. *n* = 6 mice.

Astrocyte Ca^2+^ elevation can modulate the activity of neuronal networks^42,78–80^, which may underlie the astrocyte Ca^2+^ elevation-induced sleep–wake modulation in the BF. We thus examined the changes in the synaptic activity in BF brain slices using patch-clamp recording following acute hM_3_Dq-induced astrocyte Ca^2+^ elevation in the BF in vivo^36^ (Fig. 7a–c and Supplementary Fig. S11). We found that, compared with the control group (brain slices from mice injected with GfaABC_1_D-tdTomato), the hM_3_Dq group showed significant increases in both amplitude and frequency of the miniature inhibitory postsynaptic currents (mIPSCs) (amplitude: **P = 0.005, Student’s *t-*test; frequency: ***P < 0.001, Wilcoxon rank-sum test) and the amplitude but not the frequency of miniature excitatory postsynaptic currents (mEPSCs) (amplitude: *P = 0.041, Student’s *t-*test; frequency: *P* = 0.48, Wilcoxon rank-sum test) (Fig. 7b and Supplementary Fig. S11a, c), suggesting that astrocyte Ca^2+^ elevation in the BF increased neuronal transmissions of the local neural network.

We next measured the tonic inhibition of BF neurons following hM_3_Dq-induced astrocyte Ca^2+^ elevation in the BF in vivo, because astrocytes are known to regulate ambient GABA levels^36,81–83^. GABA tonic inhibition was measured by bath application of GABA_A_ receptor antagonist (SR95531, 20 µM) and recording the changes in holding current at −70 mV^36^ (Supplementary Fig. S11b). Tonic inhibition was significantly larger in mice with hM_3_Dq-induced astrocyte Ca^2+^ elevation in the BF, compared with that from the control group (CNO treatment in mice injected with AAVs expressing GfaABC_1_D-tdTomato) (Fig. 7c; **P = 0.0011, Wilcoxon rank-sum test). Together, these results show that astrocyte Ca^2+^ elevation in the BF can cause various modulations to the local neural network, which may then produce the modulation of the sleep–wake cycle.

Our previous work shows that activation of VGLUT2^+^ neurons in the BF causes a large increase in extracellular adenosine levels. Therefore, our data that activation of astrocytes in the BF produced no detectable increase in adenosine level suggested that astrocyte activation cannot cause a large increase in the activity of VGLUT2^+^ neurons. To test this, we optogenetically activated BF astrocytes and measured the Ca^2+^ signal from VGLUT2^+^ neurons (Supplementary Fig. S12). As predicted, we found no significant change in the activity of VGLUT2^+^ neurons (Supplementary Fig. S12b, c, *P* = 0.09. Student *t* test). Together, these results suggest that astrocyte activity plays a regulatory role in local neuronal networks; however, such modulation may not lead to substantial changes in neuronal activity.

### Astrocyte Ca^2+^ activity in the BF mainly depends on local neuronal activity

Next, we studied the neural mechanisms that caused the astrocyte Ca^2+^ transients in the BF^42,84^. It has been reported that norepinephrine (NE) released by locus coeruleus (LC) can induce prominent Ca^2+^ activity in astrocytes in multiple brain regions^42,50,60,85–88^. However, because we observed a high level of astrocyte Ca^2+^ activity during both wakefulness and REM sleep, and given the fact that LC NE signaling is largely reduced during REM sleep^89^, we thus reasoned that NE signaling might not be a major factor in causing the astrocyte Ca^2+^ transients in the BF during the sleep–wake cycle.

To test this idea, we used prazosin (i.p. injection) to block NE α1 receptors, which are responsible for the NE-induced astrocyte Ca^2+^ signals^50,86,88^, and measured the changes in astrocyte Ca^2+^ signal in the BF using fiber photometry recording of GCaMP fluorescence in vivo (Supplementary Fig. S13a, b). We found no significant differences in astrocyte Ca^2+^ activity after prazosin application (Supplementary Fig. S13b; *P* = 0.91, Wilcoxon signed-rank test), suggesting that NE is not an important source of the astrocyte Ca^2+^ in the BF.

Another major neuromodulator known to induce Ca^2+^ elevation in astrocytes is acetylcholine (Ach), acting through muscarinic Ach receptors^42,90–92^, and Ach is also a major neuromodulator in the BF^93^. We thus tested the contribution of Ach by blocking the muscarinic Ach receptors using atropine (i.p. injection) and found no significant changes in the GCaMP signals after drug injection in vivo (Supplementary Fig. S13c, d; *P* = 0.43, Paired *t-*test).

Lastly, we examined the contribution of local neuronal activity in elevating astrocyte Ca^2+^ signals. We suppressed BF neuronal activity by local infusion of muscimol via an implanted cannula and found that astrocyte Ca^2+^ activity was substantially reduced (Supplementary Fig. S13e, f; ***P < 0.001, Student’s *t-*test). Together, these results suggest that local neural activity is the major contributor to the astrocyte Ca^2+^ activity recorded during the sleep–wake cycle.

This conclusion was further supported by our direct recording of astrocyte Ca^2+^ activity (using GCaMP) and multi-unit neuronal activity in the BF (Fig. 7d–h).The two signals had highly correlated dynamics in both amplitude and time course (Fig. 7f, h; Pearson’s *r* = 0.70, ***P < 0.001).

There are multiple cell types in the BF^19,93,94^, including cholinergic neurons, glutamatergic neurons (expressing vesicular glutamate transporter 2, VGLUT2), and GABAergic neurons (expressing vesicular GABA transporter, VGAT), playing distinct roles in the sleep–wake regulation^18,19,95^. We thus further examined the contribution of these different types of neurons to the observed Ca^2+^ increase in astrocytes during the sleep–wake cycle. Given that we found no critical involvement of the cholinergic muscarinic receptor signaling, we thus focused on the contribution of VGLUT2^+^ neurons and VGAT^+^ neurons.

We first examined the correlation between the Ca^2+^ activity of BF astrocytes and VGLUT2^+^ neurons or VGAT^+^ neurons via simultaneous fiber photometry recording of GCaMP signals using the bilateral dual-probe method in the VGLUT2-Cre or VGAT-Cre mice^96^, respectively (Fig. 7i and Supplementary Fig. S14a, b). We found that both VGLUT2^+^ neurons and VGAT^+^ neurons showed highly correlated Ca^2+^ activity with that in the BF astrocytes (Fig. 7j, k and Supplementary Fig. S14c–h; VGLUT2^+^ neurons vs BF astrocytes: Pearson’s *r* = 0.76, ***P < 0.001; VGAT^+^ neurons vs BF astrocytes: Pearson’s *r* = 0.80, ***P < 0.001), and the time difference of astrocyte Ca^2+^ events and the neuronal Ca^2+^ events for both VGLUT2^+^ neurons and VGAT^+^ neurons was close to zero, suggesting that both types of neurons may contribute to the increase of astrocyte Ca^2+^ in the BF.

Notably, we have observed little difference in the rising phase between the population Ca^2+^ signals from astrocytes and neurons in the BF, and this could be due to the limited temporal resolution (1 Hz) of our fiber photometry recording, although the previous study has reported that astrocytes can exhibit rapid Ca^2+^ signal on the timescale similar to neurons^97^. Future studies with single-cell resolution and faster temporal resolution will be needed to further characterize the activity of astrocytes and neurons in the BF.

To further confirm the contribution of these two types of neurons, we optogenetically activated VGLUT2^+^ or VGAT^+^ neurons in the BF using ChrimsonR and measured the evoked astrocyte Ca^2+^ level via fiber photometry recording of GCaMP signals (Fig. 7l and Supplementary Fig. S14i–l). We found that activation of both types of neurons could cause a laser frequency-dependent increase in astrocyte Ca^2+^ level (Fig. 7m, n). Such increase was not caused by non-specific effects of the laser stimulation (e.g., local heating), because laser application in mice without ChrimsonR evoked no detectable change in the astrocyte Ca^2+^ signal (Supplementary Fig. S7f–h). Together, these results suggest that cell type-specific local neuronal activity in the BF plays a major role in controlling the astrocyte Ca^2+^ activity.

### Regulation of sleep–wake cycle by astrocytes in the brainstem

Astrocytes in different brain regions exhibit significant heterogeneity^6,98^. We thus further examined whether astrocyte Ca^2+^ elevation in other brain regions could also regulate the sleep–wake cycle through similar mechanisms as we observed in the BF.

**Fig. 8:**
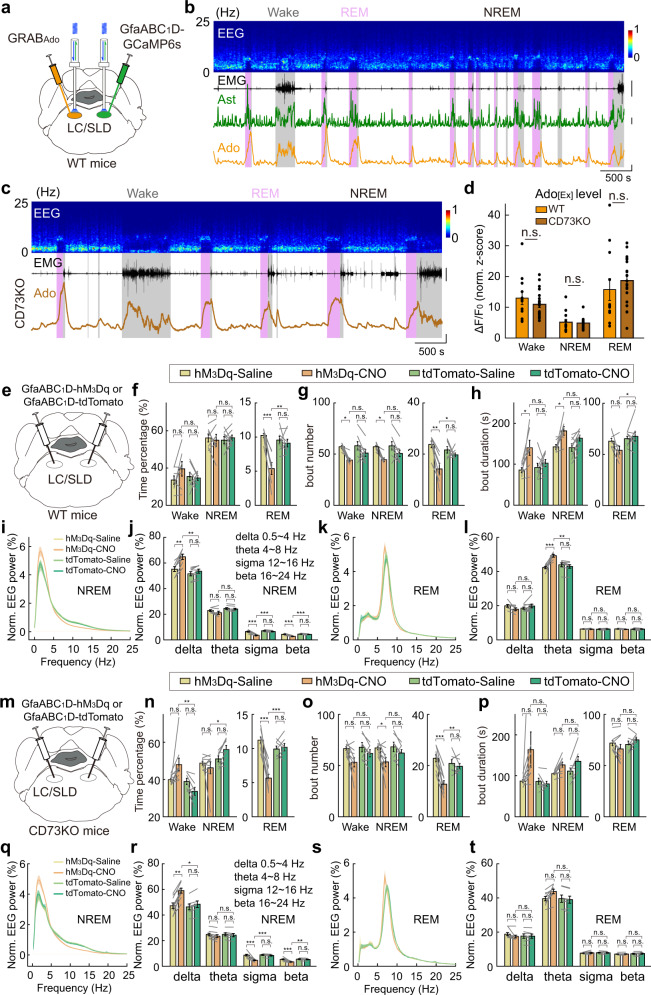
Sleep–wake regulation by astrocytes in the brainstem. **a** Schematic diagram depicting fiber photometry recording of extracellular adenosine level and population Ca^2+^ signal of astrocytes in LC/SLD region of the brainstem during the sleep–wake cycle. **b** Top to bottom, EEG power spectrogram, EMG (scale, 1 mV), GCaMP fluorescence (scale, 1 *z*-score), and GRAB_Ado_ fluorescence (scale, 1 *z*-score). **c** Fiber photometry recording of extracellular adenosine level in LC/SLD in CD73-KO mice during the sleep–wake cycle. **d** GRAB_Ado_ signal in WT and CD73-KO mice in different brain states. Each line represents data from one recording. *n* = 12 sessions from 5 mice in the WT group; *n* = 20 sessions from 6 mice in the CD73-KO group. Wake: *P* = 0.52; NREM: *P* = 0.47; REM: *P* = 0.22; Wilcoxon rank-sum test. **e**–**l** Same as Fig. 3**g**–**n**, respectively, except that experiments were performed in LC/SLD. hM_3_Dq group, *n* = 7 mice; tdTomato group, *n* = 8 mice. The statistical method used was two-way repeated measures ANOVA, followed by Tukey’s post hoc multiple comparison test. In **f**, hM_3_Dq-CNO vs tdTomato-CNO: Wake, *P* = 0.47; NREM, *P* = 0.97; REM, **P = 0.0024. hM_3_Dq-Saline vs hM_3_Dq-CNO: Wake, *P* = 0.34; NREM, *P* = 0.98; REM, ***P < 0.001. tdTomato-Saline vs tdTomato-CNO: Wake, *P* = 0.99; NREM, *P* = 0.97; REM, *P* = 0.94. In **g**, hM_3_Dq-CNO vs tdTomato-CNO: Wake, *P* = 0.35; NREM, *P* = 0.42; REM, *P = 0.045. hM_3_Dq-Saline vs hM_3_Dq-CNO: Wake, *P = 0.030; NREM, *P = 0.034; REM, **P = 0.0011. tdTomato-Saline vs tdTomato-CNO: Wake, *P* = 0.27; NREM, *P* = 0.25; REM, *P* = 0.70. In **h**, hM_3_Dq-CNO vs tdTomato-CNO: Wake, *P* = 0.12; NREM, *P* = 0.41; REM, *P = 0.045. hM_3_Dq-Saline vs hM_3_Dq-CNO: Wake, *P = 0.021; NREM, *P = 0.021; REM, *P* = 0.28. tdTomato-Saline vs tdTomato-CNO: Wake, *P* = 0.88; NREM, *P* = 0.19; REM, *P* = 0.95. In **j**, hM_3_Dq-CNO vs tdTomato-CNO: delta, **P = 0.0021; theta, *P* = 0.088; sigma, ***P < 0.001; beta, ***P < 0.001. hM_3_Dq-Saline vs hM_3_Dq-CNO: delta, **P = 0.0085; theta, *P* = 0.46; sigma, ***P < 0.001; beta, ***P < 0.001. tdTomato-Saline vs tdTomato-CNO: delta, *P* = 0.81; theta, *P* = 0.97; sigma, *P* = 0.53; beta, *P* = 0.58. In **l**, hM_3_Dq-CNO vs tdTomato-CNO: delta, *P* = 0.38; theta, **P = 0.0013; sigma, *P* = 0.98; beta, *P* = 0.89. hM_3_Dq-Saline vs hM_3_Dq-CNO: delta, *P* = 0.36; theta, ***P < 0.001; sigma, *P* = 0.97; beta, *P* = 0.73. tdTomato-Saline vs tdTomato-CNO: delta, *P* = 0.67; theta, *P* = 0.84; sigma, *P* = 0.93; beta, *P* = 1.0. **m**–**t** Same as **e**–**l**, respectively, except that experiments were performed in CD73-KO mice. hM_3_Dq group, *n* = 9 mice; tdTomato group, *n* = 7 mice. The statistical method used was two-way repeated measures ANOVA, followed by Tukey’s post hoc multiple comparison test. In **n**, hM_3_Dq-CNO vs tdTomato-CNO: Wake, **P = 0.0020; NREM, *P = 0.023; REM, ***P < 0.001. hM_3_Dq-Saline vs hM_3_Dq-CNO: Wake, *P* = 0.064; NREM, *P* = 0.80; REM, ***P < 0.001. tdTomato-Saline vs tdTomato-CNO: Wake, *P* = 0.40; NREM, *P* = 0.40; REM, *P* = 0.98. In **o**, hM_3_Dq-CNO vs tdTomato-CNO: Wake, *P* = 0.32; NREM, *P* = 0.34; REM, **P = 0.0077. hM_3_Dq-Saline vs hM_3_Dq-CNO: Wake, *P* = 0.051; NREM, *P = 0.050; REM, ***P < 0.001. tdTomato-Saline vs tdTomato-CNO: Wake, *P* = 0.68; NREM, *P* = 0.67; REM, *P* = 0.93. In **p**, hM_3_Dq-CNO vs tdTomato-CNO: Wake, *P* = 0.098; NREM, *P* = 0.80; REM, *P* = 0.23. hM_3_Dq-Saline vs hM_3_Dq-CNO: Wake, *P* = 0.11; NREM, *P* = 0.12; REM, *P* = 0.53. tdTomato-Saline vs tdTomato-CNO: Wake, *P* = 1.0; NREM, *P* = 0.13; REM, *P* = 0.77. In **r**, hM_3_Dq-CNO vs tdTomato-CNO: delta, *P = 0.015; theta, *P* = 0.95; sigma, ***P < 0.001; beta, **P = 0.0015. hM_3_Dq-Saline vs hM_3_Dq-CNO: delta, **P = 0.0042; theta, *P* = 0.81; sigma, ***P < 0.001; beta, ***P < 0.001. tdTomato-Saline vs tdTomato-CNO: delta, *P* = 0.91; theta, *P* = 0.98; sigma, *P* = 0.90; beta, *P* = 0.96. In **t**, hM_3_Dq-CNO vs tdTomato-CNO: delta, *P* = 1.0; theta, *P* = 0.18; sigma, *P* = 1.0; beta, *P* = 0.85 hM_3_Dq-Saline vs hM_3_Dq-CNO: delta, *P* = 0.72; theta, *P* = 0.22; sigma, *P* = 0.95; beta, *P* = 1.0. tdTomato-Saline vs tdTomato-CNO: delta, *P* = 1.0; theta, *P* = 0.99; sigma, *P* = 0.99; beta, *P* = 0.99.

In the LC/sublaterodorsal nucleus (SLD) region of the brainstem, which is critical for REM sleep regulation^99,100^, we found that astrocyte Ca^2+^ activity was significantly increased during both wakefulness and REM sleep (Fig. 8a, b and Supplementary Fig. S15a), exhibited rapid changes during brain state transitions (Supplementary Fig. S15b), and was highly correlated with extracellular adenosine levels (Fig. 8a, b and Supplementary Fig. S16), similar to that in the BF. However, the CD73-KO mice showed a comparable level of extracellular adenosine with that in WT mice (Fig. 8c, d; Wake, *P* = 0.52; NREM, *P* = 0.47; REM, *P* = 0.22; Wilcoxon rank-sum test), and the hM_3_Dq-induced astrocyte Ca^2+^ elevation caused similar modulation to the sleep–wake behavior in CD73-KO mice and WT mice (Fig. 8e–t and Supplementary Figs. S17 and S18). The most prominent change in the sleep–wake cycle was a reduction in the total time of REM sleep in the hM_3_Dq group (Fig. 8f, n; WT was reduced by 41%, **P = 0.0024; CD73-KO was reduced by 44%, ***P < 0.001). Also diverse modulations to the NREM EEG-Delta power was increased while high-frequency oscillations, including sigma and beta bands, were reduced (Fig. 8j, r; **P = 0.0021, ***P < 0.001, ***P < 0.001 for delta, sigma, and beta for WT mice; *P = 0.015, ***P < 0.001, **P = 0.0015 for CD73-KO mice). These results showed that astrocyte Ca^2+^ elevation in the brainstem might not contribute to extracellular adenosine through the classically proposed ATP-adenosine pathway mediated by CD73, providing further evidence supporting adenosine-independent modulation of the sleep–wake behavior by astrocytes.

Although hM_3_Dq-induced astrocyte Ca^2+^ elevation in the LC/SLD also reduced REM sleep as that found in the BF, it appears that this effect was not caused by fragmented NREM sleep because there was no statistical difference in either NREM bout number or bout duration (Fig. 8g, h; bout number, *P* = 0.42; bout duration, *P* = 0.41). Furthermore, activation of astrocytes in the two brain regions produced opposite modulation of NREM EEG (Figs. 8j and 3l), reducing delta power in the BF but increasing delta power in the LC/SLD. The latter might be caused by the more significant wake-promoting effect of astrocyte activation in the LC/SLD, as shown in the time course plot after CNO injection (Supplementary Figs. S3, S9, and S17–S19). Together, these results suggest that astrocyte Ca^2+^ elevation contributes to the sleep–wake behavior through the regulation of the surrounding neural network, thus exhibiting brain region-specific modulations to the sleep–wake cycle^101^.

## Discussion

Using fiber photometry recording of astrocyte Ca^2+^ transients and dynamics in the level of extracellular adenosine and ATP, together with chemogenetic manipulations, optogenetic manipulations, and transgenic mouse model, we provided multiple lines of evidence supporting that astrocytes can modulate the sleep–wake behavior independent of adenosine signaling pathways. Although astrocyte Ca^2+^ transients showed brain state-dependent changes during the sleep–wake cycle (Fig. 2) and had a strong correlation with extracellular adenosine level (Fig. 4b–e), optogenetically evoked Ca^2+^ elevation in BF astrocytes caused no detectable adenosine increase (Supplementary Fig. S6). In the two brain regions critical for sleep–wake control, after genetic deletion of an essential enzyme mediating extracellular ATP-adenosine conversion, there was no detectable effect on the level of extracellular adenosine (Figs. 5a, b and 8c, d), and elevation of astrocyte Ca^2+^ by chemogenetically activating astrocyte Gq pathway could still significantly modulate the sleep–wake behavior (Figs. 3g–n, 5h–o, and 8e–t). Additionally, directly suppressing astrocyte Ca^2+^ activity in the BF by conditional KO of the IP_3_R_2_ receptors caused significant modulation of the sleep–wake cycle (Fig. 3s, t and Supplementary Fig. S4c–f); however, no detectable change in the extracellular level of adenosine was observed (Fig. 6e, f). Together, these results strongly suggested that astrocytes could modulate the sleep–wake cycle independent of adenosine signaling pathways.

Astrocytes have long been thought to play a role in sleep–wake regulation^3,7^. Astrocyte Ca^2+^ activity in multiple brain regions (e.g., cortex and hippocampus) has been reported to show prominent changes during the sleep–wake cycle^51–54^, and whole animal global reduction of the astrocyte Ca^2+^ activity via genetic ablation of IP_3_R_2_^51^ or the stromal interaction molecule 1 (STIM1)^53^ altered the sleep–wake behavior. In the current study, we have recorded and manipulated the dynamics of astrocyte Ca^2+^ activity in brain regions that are directly involved in sleep–wake control, providing new data to dissect the cellular mechanisms of sleep–wake modulation by astrocytes.

In the BF, the astrocyte Ca^2+^ activity was primarily generated by local neuronal activity (Supplementary Fig. S13e, f) but not by neuromodulatory signaling such as NE or Ach (Supplementary Fig. S13a–d), different from that in other brain regions^42,50,60,80,84,86,87,90–92^ or in zebrafish^88^. Such local neuronal activity-dependent astrocyte activity caused a close correlation between neuronal and astrocyte activities (Fig. 7j, k and Supplementary Fig. S14c–h). We have also demonstrated that the Ca^2+^ elevation in BF astrocytes could produce various feedback modulations to the local synaptic transmissions (Fig. 7b, c and Supplementary Fig. S11), which may underlie the astrocyte Ca^2+^ elevation-induced sleep–wake modulation.

The BF is a well-documented brain region that plays a critical role in sleep–wake regulation^16,18,19^, and neurons in the BF are primarily GABAergic neurons^102,103^ that are more active during wakefulness than during sleep^19,104^. Our finding of the local neuronal activity-dependent astrocyte activity and the feedback modulation of the local neuronal network by astrocytes suggested that BF astrocytes may amplify the activation of the BF neural network. Because activation of BF neurons mainly promotes wakefulness, thus, the above notion can also explain our results that Ca^2+^ elevation in BF astrocytes decreased the consolidation of NREM sleep (Fig. 3h–n) and suppressing astrocyte Ca^2+^ activity caused more sleep (Fig. 3s, t and Supplementary Fig. S4c–f).

We would like to note that in our chemogenetic manipulation experiments, the careful experimental design by applying multiple pre-habituation trials to the mice and interleaved, multiple experiment sessions for control and CNO injections revealed that low doses of CNO (0.5–1 mg/kg) constantly caused a moderate increase of NREM sleep and decrease of wakefulness in the control group (Figs. 3h, 5i, and 8f, n), a known side effect of CNO^57,58^ that has been largely underestimated in the literature. However, multiple lines of our evidence supported that the major effect that we observed in the experimental groups was likely caused by Ca^2+^ elevation in astrocytes rather than non-specific side effects of CNO: First, CNO-induced modulation in the control groups were generally minor and showed opposite effects compared to the hM_3_Dq group; second, the main effect of the chemogenetic manipulation in the hM_3_Dq groups were reducing REM sleep, which was not observed in the control groups; third, the use of two-way ANOVA can separately evaluate the contribution of CNO and CNO-induced astrocyte Ca^2+^ elevation, determining the actual effects of astrocyte activation. Moreover, the role of BF astrocytes in sleep–wake regulation was also supported by our loss-of-function manipulation, in which we suppressed the astrocyte Ca^2+^ elevation by the knockout of the IP_3_R_2_ receptors.

It has been believed that adenosine signaling pathways play a critical role in the sleep–wake modulation by astrocytes, primarily based on studies using the dnSNARE mice to globally reduce exocytosis in astrocytes and indirect evaluation of the contribution of extracellular adenosine^3,7,8,105^. However, in these studies, because both neuronal transmissions and gliotransmission of the dnSNARE mice were suppressed by the leaky expression of dnSNARE that was mediated by constitutive GFAP promoter, their observed modulation of sleep–wake behavior cannot be simply attributed to reduced exocytosis in astrocytes. Indeed, decreased neuronal exocytosis could explain the alternation of EEG slow-wave oscillations and the regulation of sleep homeostasis in dnSNARE mice. In our study, using highly specific transgene expressions in astrocytes mediated by AAV5 and GfaABC_1_D promoter^35,36^ to bidirectionally manipulate astrocyte Ca^2+^ activity, we demonstrated that brain region-specific astrocyte Ca^2+^ elevation plays a role in regulating the sleep–wake cycle. Importantly, we also measured extracellular levels of ATP and adenosine using genetically encoded novel GRAB sensors while manipulating factors associated with astrocyte-derived adenosine. Removing one of the key enzymes, CD73, that mediates ATP-to-adenosine conversion caused no significant change in adenosine levels in both BF and brain stem, indicating that the CD73-mediated classical pathway does not have a major role in elevating extracellular adenosine levels. However, since other ectoenzymes may also convert ATP to adenosine and the brain region-specific regulation of this pathway is imcompletely understood^106^, we further examined the contribution of astrocyte-derived ATP by manipulating the intracellular Ca^2+^ levels that control ATP exocytosis. We suppressed astrocyte Ca^2+^ elevation through conditional KO of the IP_3_R_2_ receptors and found that such manipulation only reduced the amount of ATP but not adenosine. This result strongly suggests that astrocytes have limited contribution in elevating the extracellular level of adenosine^17,75^ in a brain region important for sleep–wake regulation, further indicating that astrocytes can regulate the sleep–wake cycle independent of adenosine signaling pathways. Together, these results provide new insights into the diverse mechanisms in the modulation of the sleep–wake cycle by astrocyte activity. Furthermore, these results provide evidence supporting that neurons, rather than astrocytes, are the primary source of extracellular adenosine^16,17,75^

In the current study, our measurements of astrocyte Ca^2+^ activity and the extracellular dynamics of adenosine/ATP were all based on fiber photometry recording, which reports the population signal. Thus, an important question raised by our results is how these activities are spatially organized and related to each other, especially in microdomains of astrocytes^37,107^. Moreover, we have provided multiple pieces of evidence that strongly suggest that astrocytes may not contribute critically to elevating the extracellular adenosine level. Thus, they could regulate sleep through adenosine-independent pathways. However, considering the significant heterogeneity of astrocytes in different brain regions^6,98^, future studies are needed to investigate whether our current findings also apply to other brain regions. Lastly, although our current findings significantly challenge the previous theory that adenosine signaling is key to astrocyte regulation of the sleep–wake cycle, future studies with a complete blockade of astrocyte-derived ATP or other astrocyte sources of adenosine may be needed to further validate our current findings. For example, astrocytes may produce adenosine through IP_3_R_2_-insensitive ATP release, as IP_3_R_2_-KO mice still have remaining Ca^2+^ transients in astrocyte processes^60^, which may be important for adenosine generation; astrocytes may also release adenosine directly via transmembrane transporters^108^; it is also possible to have Ca^2+^-independent ATP or adenosine release from astrocytes. However, a technical challenge with the complete removal of astrocyte sources of ATP or adenosine is that these manipulations may cause significant abnormality in astrocytes, as both ATP and adenosine are important components of the purinergic pathway that are essential for cell metabolism and survival^109^. In addition to these limitations, future studies are also needed to address the alternative cellular mechanisms that underlie astrocyte modulation of the neuronal network in the BF and in other brain regions that are essential for sleep–wake regulation.

## Materials and methods

### Mice

All experimental procedures have followed the guidelines of the National Institutes of Health and approved by the Animal Care and Use Committee at the Institute of Neuroscience, Chinese Academy of Sciences. Brain slice experiments were performed using young mice (only male mice were used, ~3 weeks at the time of virus injection). In vivo experiments were performed on adult mice (including both genders, > 6 weeks at the time of data collection). Mice were housed in a 12/12-h light/dark cycle (light on at 7 AM) with food and water available ad libitum. Mice with implants for EEG/EMG recording, optogenetic manipulation, or fiber photometry recording were housed individually. WT mice (C57BL/6) were purchased from institute-approved vendors (Shanghai Silaike or LingChang Experiment Animal Co., China). VGAT-IRES-Cre (*Slc32a1*^*tm2(cre)Lowl*^/J, #016962), VGLUT2-IRES-Cre (*Slc17a6*^*tm2(cre)Lowl*^/J, # 016963) and CD73-KO mice (B6.129S1-*Nt5e*^*tm1Lft*^/J, #018986), GFAP-Cre/ERT2 mice (B6.Cg-Tg(GFAP-cre/ERT2)505Fmv/J, #012849), GFAP-Cre (FVB-Tg(GFAP-cre)25Mes/J, #004600), Aldh1l1-Cre (B6;FVB-Tg(Aldh1l1-Cre)JD1884Htz/J, #023748) were obtained from The Jackson Laboratory. IP_3_R_2_^*flox*^ mice were kindly shared by Kunfu Ouyang from Peking University. GFAP-Cre mice were back-crossed to C57BL/6J mice for at least four generations to reduce the FVB strain contribution before usage.

### Surgical procedures

All surgical procedures were conducted under general anesthesia using continuous isoflurane (5% for induction; 1.5%–2% for maintenance). Depth of anesthesia was monitored continuously and adjusted when necessary. Following induction of anesthesia, the mice were placed on a stereotaxic frame with a heating pad.

To implant EEG and EMG electrodes, two stainless steel screws for EEG were inserted into the skull above the visual cortex, two other screws were inserted into the skull above the frontal cortex, two insulated EMG electrodes were inserted into the neck muscle, and a reference electrode was attached to a screw inserted into the skull above the cerebellum.

For experiments with virus injection, a craniotomy (~0.5 mm in diameter) was made on top of the BF or LC/SLD, and virus (0.2–0.4 µL) was injected into the BF (AP 0 mm, ML 1.3 mm, DV 5.0 mm from the cortical surface) or LC/SLD (AP –5.6 mm, ML 0.9 mm, DV 3.9 mm from bregma) using Nanoject II (Drummond Scientific) via a glass pipette (23 nL/injection, inter-injection interval 15–30 s). The following AAV viruses were used in current study: AAV2/9-hSyn-GRAB_Ado1.0_ (Addgene vector #153285; titer: 1.5 × 10^13^ or 4.7 × 10^13^ vg/mL; Vigene Biosciences); AAV2/9-GfaABC_1_D-GRAB_Ado1.0_ (Addgene vectors #153288; titer: 5 × 10^12^ vg/mL; Vigene Biosciences); AAV2/5-GfaABC_1_D-cyto-GCaMP6f (Addgene vector #52925; titer: 1.8 × 10^13^ vg/mL; Vigene Biosciences); AAV2/9-GfaABC_1_D-GRAB_ATP1.0_ (Addgene vectors #167579; titer: 2.4 × 10^12^ vg/mL; BrainVTA Co., China); AAV2/5-GfaABC_1_D-hM3Dq-mCherry (Addgene vector #92284; titer: 4.8 × 10^13^ vg/mL; Vigene Biosciences); AAV2/5-GfaABC_1_D-tdTomato (Addgene vector #44332, titer: 2.2 × 10^13^ vg/mL; Vigene Biosciences); AAV2/5-GfaABC_1_D-ChrimsonR-tdTomato (the plasmid was constructed by Yulong Li’s lab; titer: 3 × 10^13^ vg/mL; Vigene Biosciences); AAV2/9-EF1α-DIO-GCaMP6f (titer: 2 × 10^12^ vg/mL, BrainVTA Co., China); AAV2/9-hSyn-FLEX-ChrimsonR-tdTomato (titer: 1 × 10^13^ vg/mL, Shanghai Taitool Bioscience Co., China); AAV2/5-GfaABC_1_D-cyto-GCaMP6s(gift from Zhifeng Liang’s Lab, Titer: 1.8 × 10^13^ vg/mL; Shanghai Taitool Bioscience Co., China); AAV2/5-GfaABC_1_D-Cre (titer: 5 × 10^12^ vg/mL, BrainVTA Co., China); AAV2/9-EF1a-DIO-hM3Dq-mCherry (titer: 5 × 10^12^ vg/mL, BrainVTA Co., China); AAV2/9-CAG-DIO-hM3Dq-mCherry (titer: 5.2 × 10^12^ vg/mL, BrainVTA Co., China); AAV2/9-EF1a-DIO-GCaMP6s (titer: 5.2 × 10^12^ vg/mL, BrainVTA Co., China); AAV2/8-GfaABC_1_D-cyto-GCaMP6m (titer: 1 × 10^13^ vg/mL, Shanghai Taitool Bioscience Co., China).

For all the brain slice experiments, AAVs expressing GfaABC_1_D-hM3Dq were injected into one hemisphere of the BF, and AAVs expressing GfaABC_1_D-tdTomato were injected into the contralateral hemisphere of the BF.

For fiber photometry recording or optogenetic manipulation experiments, a craniotomy (0.5–1 mm in diameter) was made 1–2 weeks after virus injection, and one or two optical fibers (200 µm in diameter, Thorlabs) with FC ferrule were inserted into the BF or SLD using the same coordinate for virus injection.

For extracellular recording experiments, stereotrodes carried by a lightweight driver were implanted into the BF as described previously^19^. The stereotrodes were made by twisting two micro-wires (FeNiCr wires, 25 µm in diameter, Stablohm 675, California Fine Wire), and the stereotrodes were cut with sharp scissors and electroplated with platinum (H_2_PtCl_6_, Sigma-Aldrich) to an impedance of ~300 kΩ with a lab-made MSP430 MCU-controlled multi-channel plating device.

For the muscimol infusion experiment, an opto-fluid cannula assembly, which allows for precise drug infusion during fiber photometry recording, was implanted into the BF. The cannula consists of two parts, an optical fiber (200 µm in diameter, Thorlabs) with FC ferrule and an infusion cannula (200 µm in diameter). The optical fiber and the cannula were glued on a printed circuit board in parallel (500 µm apart), and the tip of the optical fiber was ~500 µm longer than that of the cannula.

All the implant was secured to the skull using dental cement.

Experiments were carried out at least one week after the last surgery.

### Polysomnography recordings

For polysomnography recordings with simultaneous fiber photometry recording or extracellular recording, mice were connected with 1 or 2 optical fibers and a flexible cable for EEG/EMG/multiunit activity. Recordings were made in the home cage placed in a sound-attenuation box (68 cm × 62 cm × 74 cm). Each recording session was started after ~30 min of habituation and lasted for 1–3 h. The EEG/EMG signals were recorded using a TDT system-3 amplifier (RZ2 + PZ5). EEG/EMG signals were high-pass filtered at 0.5 Hz and digitized at 1525 Hz.

For polysomnography recordings, mice were transferred into recording cages placed in a sound-attenuation box (80 cm × 100 cm × 120 cm), connected to the amplifier with a flexible recording cable via a commutator. Three or four mice were recorded simultaneously in one batch, and each batch had both treatment animals and control animals to minimize potential non-specific environmental influences. Mice were habituated in the recording cage for at least three days before each recording. In the chemogenetic experiments (Figs. 3g–n, 5h–o, and 8e–t and Supplementary Figs. S3, S9, and S17–S19), mice were recorded for 4 h immediately after each injection (CNO, Tocris Bioscience #4936; 1 mg/kg or 0.5 mg/kg in 50 µL saline, i.p. injection; or saline, 50 µL, i.p. injection) starting at 1 PM each day. CNO and saline injections were performed alternately every day for 4–6 days. For the IP_3_R_2_-KO experiment (Fig. 3r–t and Supplementary Fig. S4), mice were recorded for three days, and the three-day recordings for each mouse were averaged to obtain data for the 24-h sleep–wake cycle.

### Fiber photometry recording

To record fluorescence from GCaMP, the adenosine sensor or ATP sensor, we attached an optic fiber (Thorlabs, FT200UMT) to the implanted ferrule via a ceramic sleeve and recorded emission fluorescence using fiber photometry. The photometry rig was constructed using parts from Doric Lens, including a fluorescence optical mini cube (FMC4_AE (405)_E (460–490)_F (500–550)_S), a blue LED (CLED_465), a LED driver (LED_2) and a photoreceiver (NPM_2151_FOA_FC). For recordings containing both green and GCaMP isosbestic channels, an additional LED (CLED_405) was used.

During recording, a software-controlled lock-in detection algorithm was implemented in the TDT RZ2 system using the fiber photometry “Gizmo” of the Synapse software (modulation frequency: 459 Hz for single-channel recording, and 271 or 459 Hz for the second channel in two-channel mode; low-pass filter for demodulated signal: 20 Hz, sixth order). The excitation light intensity was measured as 50–70 µW (465 nm) or 25–30 µW (405 nm) from the tip of the optical fiber. The photometry data was stored using a sampling frequency of 1017 Hz.

To minimize the auto-fluorescence of the optical fiber, we bleached the recording fiber before recordings and recorded the reading of background fluorescence intensity. The background fluorescence was subtracted from the recorded signal in subsequent analysis.

For fiber photometry recording during optogenetic activation, we used a photometry rig with a fluorescence optical mini cube (FMC5_E1 (465–480)_F1 (500–540)_E2 (555–570)_F2 (580–680)_S), a LED (CLED_465), a LED driver (LED_2) and a photoreceiver (NPM_2151_FOA_FC). A 638 nm laser was introduced into the light path of the photometry system through the F2 port of the mini cube. To minimize the laser-induced artifact in the photometry signal, we used a 40-Hz square wave to modulate the excitation LED for the photometry recording and applied the laser pulse (power: 10 mW for Fig. 7m, n and Supplementary Figs. S6 and S12; 2 mW for Supplementary Fig. S7c–h; pulse duration: 10 ms) for the optogenetic stimulation during off period of the photometry LED. The timing of the photometry LED, and laser for the optogenetic stimulation was controlled by the TDT system, which was also used for data acquisition.

Extracellular adenosine levels in the BF and Ca^2+^ activity in astrocytes and neurons show brain state-dependent changes. Thus, to record the adenosine change or Ca^2+^ change directly evoked by the activation of astrocytes or neurons, rather than the indirect adenosine release or Ca^2+^ increase induced by changes in the brain states, we measured the optogenetic-evoked adenosine signal or Ca^2+^ signal under anesthesia (maintained using isoflurane at a concentration of 1.5%; body temperature of the mouse was maintained at 37 °C).

### Extracellular recording

Mice were connected with one optical fiber and a flexible cable for EEG/EMG/multiunit activity, and recordings were made in the home cage placed in a sound-attenuation box (68 cm × 62 cm × 74 cm). The stereotrode assembly was moved forward 100 µm in the BF 10 min before each recording. Each recording was started after the mouse fell asleep and lasted for ~1 h.

The EEG/EMG signals and the extracellular signals were recorded using TDT system-3 amplifiers (RZ5 + RA16PA or RZ2 + PZ5). The extracellular signals were filtered at 0.3–8 kHz and sampled at 25 kHz for offline spike detection. Multiunit spiking signals were extracted from the raw data using Offline Sorter (Plexon), and *z*-score transformed for further analysis.

### In vivo pharmacological manipulation

To examine the contribution of acetylcholine or norepinephrine signaling to the astrocyte Ca^2+^ elevation during the sleep–wake cycle, atropine (HY-B0394, MedChemExpress LLC; 5 mg/kg in 100 µL 0.9% NaCl, i.p.) or prazosin (HY-B0193A, MedChemExpress LLC; 1 mg/kg, 10 mg/mL stock in DMSO, diluted with corn oil to 50 µL immediately before use, i.p.) was administered to mice approximately 1.5 h after the start of each recording. Recordings were not interrupted during the i.p. injection. The duration of each recording was ~3 h.

For intracranial muscimol infusion experiments, muscimol (1 mg/mL dissolved in 0.9% NaCl, 200 nL) or saline (200 nL) was infused via an implanted guide cannula. Mice were head-fixed under a dissection microscope, and a microinjection pump was used to deliver the solutions at a speed of 5 nL/s. The infusion cannula was left in place for 5 min before the withdrawal. Immediately after the procedure, mice were returned to the home cage for fiber photometry recordings. Muscimol and saline were infused alternately on different days to minimize non-specific errors (e.g., signal attenuation due to photobleaching).

To examine how chemogenetic activation modulates astrocyte Ca^2+^ signals during the sleep–wake cycle, mice were injected (i.p.) with CNO (1 mg/kg) or saline approximately 1.5 h after the start of each recording. Recordings were not interrupted during the injection and each recording lasted ~3 h.

For each mouse, the power of the excitation light used for the fiber photometry recording remained the same.

### Patch-clamp recording in brain slice

Mice were prepared for brain slice recording 2–3 weeks after virus injections as described previously^19,110–113^. Mice were first injected with CNO (1 mg/kg; dissolved in saline; i.p.), and then were deeply anesthetized with pentobarbital sodium (i.p., 100 mg/kg). After decapitation, the brain was dissected rapidly and placed in ice-cold oxygenated NMDG-HEPES solution (in mM: NMDG 93, KCl 2.5, NaH_2_PO_4_ 1.2, NaHCO_3_ 30, HEPES 20, D-glucose 25, sodium ascorbate 5, thiourea 2, sodium pyruvate 3, MgSO_4_ 10 and CaCl_2_ 0.5, *N*-acetyl-l-cysteine (NAC) 12, at pH 7.4, adjusted with HCl), and coronal slices (with a thickness of 250 µm) containing the BF were made with a vibratome (Leica VT1200S). Brain slices were recovered in oxygenated NMDG-HEPES solution at 32 °C for 10 min and then maintained in an incubation chamber with oxygenated aCSF (in mM: NaCl 124, KCl 4.5, CaCl_2_ 2, MgCl_2_ 1, NaHCO_3_ 26, NaH_2_PO_4_ 1.2, and d-glucose 10) at 25 °C for 1–4 h before recording.

Whole-cell patch-clamp recordings were made using patch pipettes with a resistance of 5–6 MΩ. The recordings were performed using a Multi-Clamp 700B amplifier and pCLAMP10.3 software (Axon Instruments, Molecular Devices). The intracellular solution used for the recording of miniature EPSCs (mEPSCs) and mIPSCs included the following (in mM): CsMeSO_3_ 125, CsCl 2, HEPES 10, EGTA 0.5, Mg-ATP 4, Na-GTP 0.3, Na_2_-phosphocreatine 10, TEA-Cl 5. The intracellular solution used for tonic GABA recording included the following (in mM): KCl 138, Mg-ATP 4, Na-GTP 0.2, Na_2_-phosphocreatine 8, EGTA 1, CaCl_2_ 0.1, HEPES 10.

For mEPSC recording, neurons were voltage-clamped at –70 mV and pre-incubated with bicuculline (20 μM) and TTX (0.5 µM) for 5 min before recordings. For mIPSC recording, neurons were voltage-clamped at +10 mV and pre-incubated with CNQX (20 μM) and TTX (0.5 µM) for 5 min before recordings. For the recording of tonic GABA currents, SR95531 (20 µM) was bath-applied in the presence of CNQX (10 µM), TTX (1 µM), and APV (25 µM).

All chemicals were purchased from Sigma unless otherwise stated.

### Ca^2+^ imaging in brain slice

Coronal slices containing BF were prepared as described above, except that mice were not injected with CNO before sacrifice. Brain slice was moved to the recording chamber and imaged using a confocal microscope (Fluoview 1000; Olympus) with a 40× water-immersion objective (N.A. = 0.8) at a frame rate of 1 Hz. CNO (1 µM, Tocris Bioscience #4936) was applied in the bath.

### Histology and immunohistochemistry

To verify the virus expression and placements of optical fibers, mice were deeply anesthetized and immediately perfused using 0.1 M phosphate-buffered saline (PBS), followed by 4% paraformaldehyde (PFA). The brain tissues were removed and post-fixed overnight in 4% PFA before dehydration in a 30% sucrose solution. Brain samples were embedded with OCT compound (NEG-50, Thermo Fisher Scientific) and cut into 50 or 40 µm sections using a cryostat (HM525 NX, Thermo Scientific). Brain sections were stored at –20 °C before further processing.

For histology examination, brain sections were washed with PBS and coverslipped using mounting media containing DAPI (H-1500, Vector Labs).

For immunostaining, brain sections were permeabilized using PBST (0.3% Triton X-100 in PBS) for 30 min and incubated with a blocking solution (5% normal goat serum or normal donkey serum) for 1 h before the incubation with a primary antibody overnight at 4 °C. The brain sections were then washed with PBS or PBST and incubated with a secondary antibody. Finally, the brain sections were washed and mounted with mounting media.

The following primary or secondary antibodies were used in the current study: chicken anti-GFP (1:500; Aves Labs GFP-1020), rat anti-mCherry (1:500; Thermo Fisher Scientific M11217), rabbit anti-S100β (1:300; Abcam ab41548), guinea pig anti-NeuN (1:1000; Millipore ABN90), rabbit anti-DsRed (1:500; Takara Bio 632496), rabbit anti-Cre (1:300; Cell Signaling Technology 15036), mouse anti-Cre (1:300; Millipore MAB3120), goat anti-guinea pig Alexa Fluor 647 (1:500; Thermo Fisher Scientific A-21450), donkey anti-chicken Alexa Fluor 488 (1:500; Jackson ImmunoResearch Labs 703-545-155), goat anti-chicken Alexa Fluor 488 (1:1000; Thermo Fisher Scientific A-11039), donkey anti-rabbit Alexa Fluor Cy3 (1:750; Jackson ImmunoResearch Labs 711-165-152), donkey anti-rabbit Alexa Fluor 488 (1:300; Thermo Fisher Scientific A-21206), donkey anti-rat Alexa Fluor 594 (1:500; Thermo Fisher Scientific A-21209), goat anti-mouse Alexa Fluor 647 (1:300; Thermo Fisher Scientific A-21235).

### Quantification and statistical analysis

#### Polysomnography recording analysis

EEG spectral analysis was carried out using a fast Fourier transform (FFT) with a frequency resolution of 0.18 Hz. The brain states were scored every 5 s semi-automatically in MATLAB and validated manually by trained experimenters. Brain states classification was performed according to established criteria^16,19^: Wakefulness was defined as desynchronized EEG and high EMG activity; NREM sleep was defined as synchronized EEG with high-amplitude delta activity (0.5–4 Hz) and low EMG activity; REM sleep was defined as high power at theta frequencies (6–10 Hz) and low EMG activity.

#### Analysis of sleep architecture after chemogenetic manipulation

To analyze changes in the sleep–wake cycle after chemogenetic activation of astrocytes, we used the 4-h polysomnography recording after each injection to calculate the time percentage, bout number, and bout duration of each brain state, and the normalized EEG power spectrum for both NREM and REM sleep. We reported the averaged results for each mouse across multiple injection sessions in Figs. 3g–n, 5h–o, and 8e–t and Supplementary Figs. S3, S9, and S17–S19.

#### Quantification of virus expression regions in chemogenetic experiments and in 24-h recording test

To quantify the brain regions of AAV-mediated gene expression in the chemogenetic experiments, we processed each brain sample using a standard histology method and imaged brain sections using a slide scanner (VS-120, Olympus). The fluorescence images (AP 0 mm for BF, AP −5.6 mm for LC/LSD) were exported and registered to the Allen Mouse Brain Atlas as described previously^110,112,113^. We then detected the raw fluorescence signal and normalized the signal using the peak fluorescence intensity of the image. The normalized signal was then averaged across each experimental group to generate the heat map in Supplementary Figs. S3a, b, S4a, b, S9a, b, S17a, and S18a.

#### Fiber photometry data analysis

To analyze the photometry data, we first binned the raw data into 1 Hz (i.e., down-sampled by 1000) and subtracted the background fluorescence. We then calculated the Δ*F*/*F*_0_ using a baseline obtained by fitting the background-subtracted data with a second-order exponential function. For fiber photometry recording with both green and the GCaMP isosbestic channels (Fig. 2), we used a MATLAB script “FastICA” to unmix GCaMP signals from physiological artifacts (https://github.com/aludnam/MATLAB/tree/master/FastICA_25).

To identify fast components or remove the slow drift in the photometry signal, we used a MATLAB script “BEADS” (cut-off frequency: adenosine and ATP, 0.00035 cycles/sample; GCaMP, 0.001 cycles/sample) (https://www.mathworks.com/matlabcentral/fileexchange/49974-beads-baseline-estimation-and-denoising-with-sparsity).

The *z*-score transformed Δ*F*/*F*_0_ was used in the following analysis: (1) The correlation between astrocyte Ca^2+^ signals and GRAB_Ado_ or GRAB_ATP_ signals (Fig. 4c, h and Supplementary Fig. S16a); (2) The correlation between GRAB_ATP_ signals and GRAB_Ado_ signals (Figs. 4m and 5e); (3) The correlation between neuronal Ca^2+^ signals or multiunit activity and astrocyte Ca^2+^ signals (Fig. 7f, j, k); (4) The optogenetic activation-evoked adenosine release or Ca^2+^ increase (Fig. 7m, n and Supplementary Figs. S6c, d, S7c–h, and S12c)

To quantify the change in the adenosine signals, ATP signals, or astrocyte Ca^2+^ signals during the sleep–wake cycle across multiple animals, we further normalized (norm. *z*-score) the *z*-score transformed signal (Δ*F*/*F*_0_) using the standard deviation of these signals during NREM sleep when there was no apparent fluctuation in the signal. The normalized Δ*F*/*F*_0_ was used for the analysis in Figs. 2c–f, 3f, q, 5b, 6c, f, and 8d and Supplementary Fig. S15.

To analyze the correlation between astrocyte Ca^2+^ activity and movement, we first selected a recording with prolonged wakefulness, and then used behavioral movies to distinguish between “Locomotion” and “Quiet wakefulness”. During the locomotion period, the mice were often walking and running, in contrast to the typical behaviors, including immobility, grooming, scratching, and sniffing during the quiet wakefulness. The duration of each segment was 10–60 s.

To quantify the change in astrocyte Ca^2+^ signals after i.p. injection of atropine or prazosin, we first extracted the GCaMP events that were larger than twice the standard deviation of the signal, and then defined the mean amplitude of these events as the size of GCaMP signals. We used the size of GCaMP signals before drug applications and 10 min after drug applications to evaluate the effects of the drugs. The same method was used to calculate the size of GCaMP signals after intracranial infusion of muscimol or saline.

To quantify the change in astrocyte Ca^2+^ signal following chemogenetic activation, we first used a MATLAB function “findpeaks” to extract GCaMP events greater than twice the standard deviation of the signal from the tdTomato-expressing BF, and calculated the ratio between events from the tdTomato-expressing BF and the corresponding events from the hM_3_Dq-expressing BF. We then calculated the average ratio before and after CNO or saline injections (2 min to 1 h post injection) for each recording. This ratio was used to quantify the CNO-induced changes.

To quantify the change in astrocyte Ca^2+^ signal following KO of IP_3_R_2_ receptors, we first used a MATLAB function “findpeaks” to extract GCaMP events greater than twice the standard deviation of the signal from the Cre-expressing BF and then used them to determine the corresponding events from the tdTomato-expressing BF. We then calculated the mean amplitude of events from the Cre-expressing BF and tdTomato-expressing BF for each brain state. Normalized amplitudes were used to assess the effect of IP_3_R_2_ KO.

We found that the fluorescence signals at the beginning of each recording (~10 min) were often subjected to a faster decline, which might be caused by photobleaching of the auto-fluorescence in the brain tissue. We thus removed data during this period in the quantification.

#### Analysis of the correlation between two signals

To analyze the correlation and time course relationship between two signals in Figs. 4c–e, h–j, m–o, 5e–g, and 7f–h, j, k and Supplementary Figs. S14e–h and S16, we selected events in the GRAB_Ado_ signal (Figs. 4c–e, m–o and 5e–g and Supplementary Fig. S16), GRAB_ATP_ signal (Fig. 4h–j), or astrocyte Ca^2+^ signal (Fig. 7f–h, j, k; Supplementary Fig. S14e–h) and defined the event onset or offset as the time when the signal increased above 10% of its peak amplitude in the rising edge or decreased under 10% of the peak amplitude in the falling edge, respectively. We used the onset or offset time as a reference to align the two signals and calculated a peri-event time histogram (PETH). The PETH was further normalized (0–1) to get the plots in Figs. 4e, j, o, 5g, and 7h and Supplementary Figs. S14f, h and S16c. We defined the C_50_ of the PETH as the time when the signal passed half of its peak amplitude and used the difference in C_50_ between the two signals to estimate the averaged difference in the time course of the two signals. The Σ_flour_. (i.e., area under a curve, AUC) of a selected event (relative to a baseline defined as the mean of the signal during the 100 s period before the onset of each event) was used to generate the scatter plot in Figs. 4c, h, m, 5e, and 7f, j, k and Supplementary Fig. S16a. Due to the difference in the time course between the two signals, we subtracted the onset and offset time of the GRAB_Ado_ events (Figs. 4c, m and 5e and Supplementary Fig. S16a), GRAB_ATP_ (Fig. 4h), or astrocyte Ca^2+^ events (Fig. 7f, j, k) by the time difference in the C_50_ of the PETH of the two signals and used the shifted onset and offset time to calculate the AUC. To estimate the non-specific correlation between the two signals, we randomly shuffled the astrocyte Ca^2+^ signals (Fig. 4d, i and Supplementary Fig. S16b), the GRAB_ATP_ signals (Figs. 4n, 5f), or the neuronal Ca^2+^ signals (Fig. 7g and Supplementary Fig. S14e, g) and calculated the AUC using the same method as in the non-shuffled condition to get the scatter plot in Figs. 4d, i, n, 5f, and 7g and Supplementary Figs. S14e, g and S16b.

#### Analysis of Ca^2+^ imaging in brain slice

Analysis of time-lapse image series was performed in MATLAB similar to that described previously^36^. We first corrected the drift of the images using a rigid-affine algorithm in ANTs (http://stnava.github.io/ANTs/) and extracted Ca^2+^ signals using CNMF-E (https://github.com/zhoupc/CNMF_E). The Ca^2+^ signal was then normalized to the global background estimated in CNMF-E and used to calculate the AUC of each Ca^2+^ event. To quantify the effect of CNO on astrocyte Ca^2+^ signal, we calculated the average size of the Ca^2+^ event during the 120 s before and after CNO application and generate data for Fig. 3c.

#### Analysis of patch-clamp recording in brain slice

The mEPSCs and mIPSCs were extracted using the Mini Analysis Program (Synaptosoft). The tonic GABA current was analyzed using the Clampfit software (Molecular Devices). We manually selected the recording period before and after the application of SR95521, and used the shift in the averaged current to calculate the tonic GABA current.

#### Statistical tests

##### Statistic procedures

We first performed a normality test on each dataset using the Shapiro–Wilk test. Parametric tests (paired or unpaired Student’s *t-*tests) were used if the dataset was normally distributed (*P* < 0.05), otherwise non-parametric tests (Wilcoxon signed-rank test or Wilcoxon rank-sum test) were used. Multiple group comparisons (Figs. 3g–n, 5h–o, and 8e–t and Supplementary Figs. S3, S9, S17–S19) were performed by two-way repeated measures ANOVA, followed by Tukey’s post hoc multiple comparison test. All the statistical tests were two-tailed and performed in MATLAB or OriginPro 2019b. The significance level was set at *P* = 0.05.

##### Sample size

We did not perform a calculation on the sample size. We used a sample size comparable to studies using similar techniques and animal models.

##### Data exclusion criteria

Mice in the chemogenetic experiments and fiber photometry experiments were excluded based on post-hoc verification of the virus expression or the placement of the optical fiber.

The investigators were not blinded to the genotypes or the experimental conditions of the animals.

## Supplementary information

SUPPLEMENTAL MATERIAL

## Data Availability

The data supporting the findings of this study are included in the figures and supporting files. The raw data are available from the corresponding author upon request.
