# Supplementary material for: Adenosine-independent regulation of the sleep–wake cycle by astrocyte activity
Source: Cell Discov. 2023 Feb 7;9:16. doi: 10.1038/s41421-022-00498-9 (PMC9902472; doi:10.1038/s41421-022-00498-9)
Supplement: Supplementary file 1 — SUPPLEMENTAL MATERIAL [file 41421_2022_498_MOESM1_ESM.pdf]

Supplementary Information for

Adenosine-Independent Regulation of the Sleep-Wake Cycle by Astrocyte Activity

Wanling Peng[1,2,9]*, Xiaotong Liu[1,2,9], Guofen Ma[4,5,9], Zhaofa Wu[6,8], Ziyue Wang[5], Xiang Fei[1,2], Meiling Qin[1], Lizhao Wang[5], Yulong Li[6,7,8], Siyu Zhang[4,5]*, and Min Xu[1,2,3]*

*Correspondence: mxu@ion.ac.cn; or zhang_siyu@sjtu.edu.cn; or wlpeng@ion.ac.cn

This PDF file includes:

Figures S1 to S19

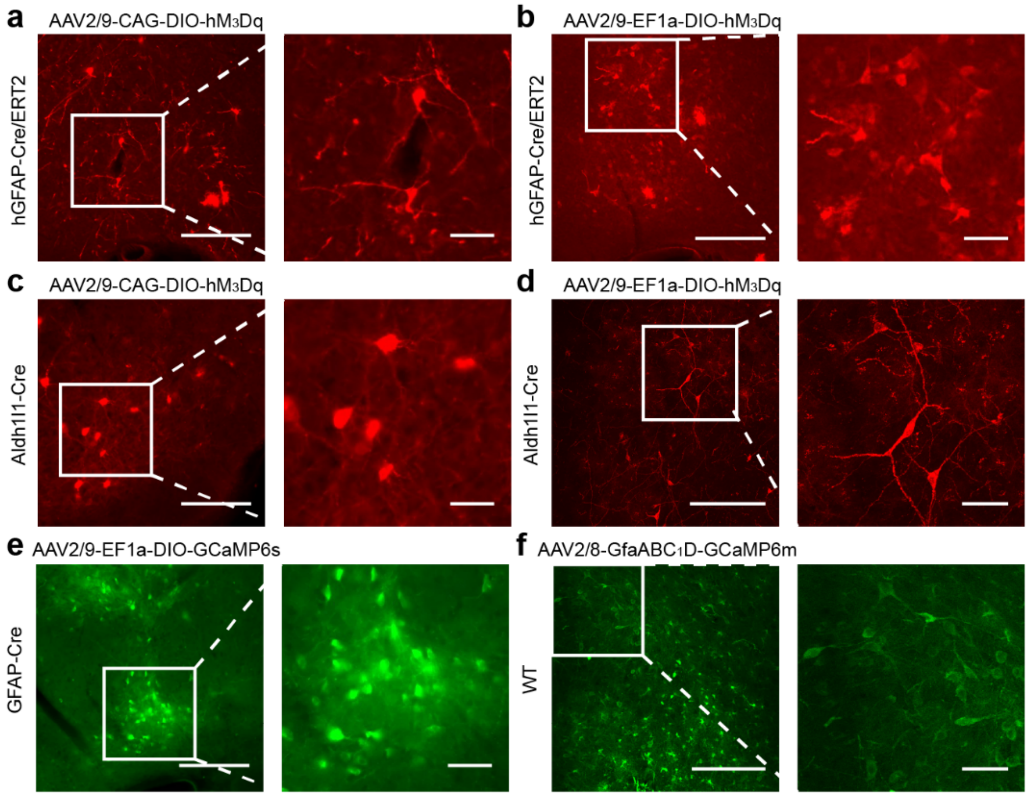

Supplementary Fig. S1 Multiple methods cause non-specific leaky expression in BF neurons (related to Fig. 1)

a-b Fluorescence images showing apparent leaky expression in BF neurons in hGFAP-Cre/ERT2 mice. **c-e** Same as **a** and **b**, except that experiments were performed using other transgenic mouse lines. **f** Fluorescence images showing apparent leaky expression in BF neurons after injecting AAV2/8-GfaABC$_1$D-GCaMP6m into WT mice. Scale bars represent 200 μm (left) and 50 μm (right) in all panels.

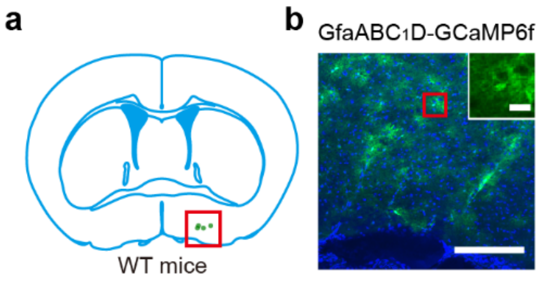

Supplementary Fig. S2 Histological verification of optic fiber position (related to Fig. 2)

a Schematic diagram summarizing placement of the optical fiber in mice expressing GfaABC$_1$D-GCaMP. **b** Fluorescence image of the BF showing the expression of the GfaABC$_1$D-GCaMP. Scale bars represent 200 μm and 20 μm (inset), respectively.

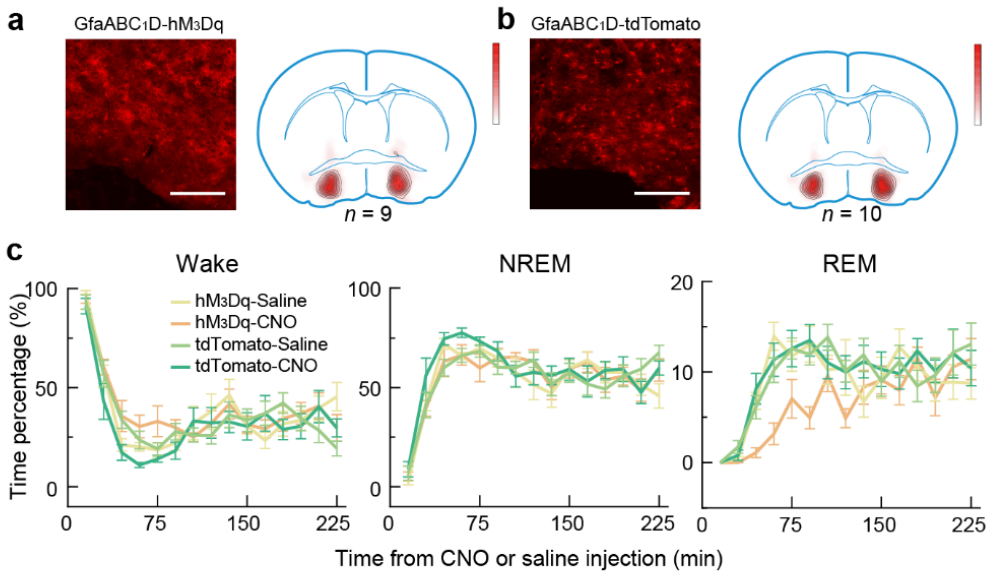

Supplementary Fig. S3 Sleep-wake modulation by chemogenetic-induced Ca^{2+} elevation in BF astrocytes in WT mice (related to Fig. 3)

a Fluorescence image of the BF showing the expression of the GfaABC₁D-hM₃Dq in the BF (left) and the normalized expression density of GfaABC₁D-hM₃Dq from 9 mice (right). Scale, 200 μm. **b** Same as **a**, except that GfaABC₁D-tdTomato was injected. tdTomato group, *n* = 10 mice. **c** Time percentage of each brain state after the injection of CNO or saline in hM₃Dq- or tdTomato-expressing mice.

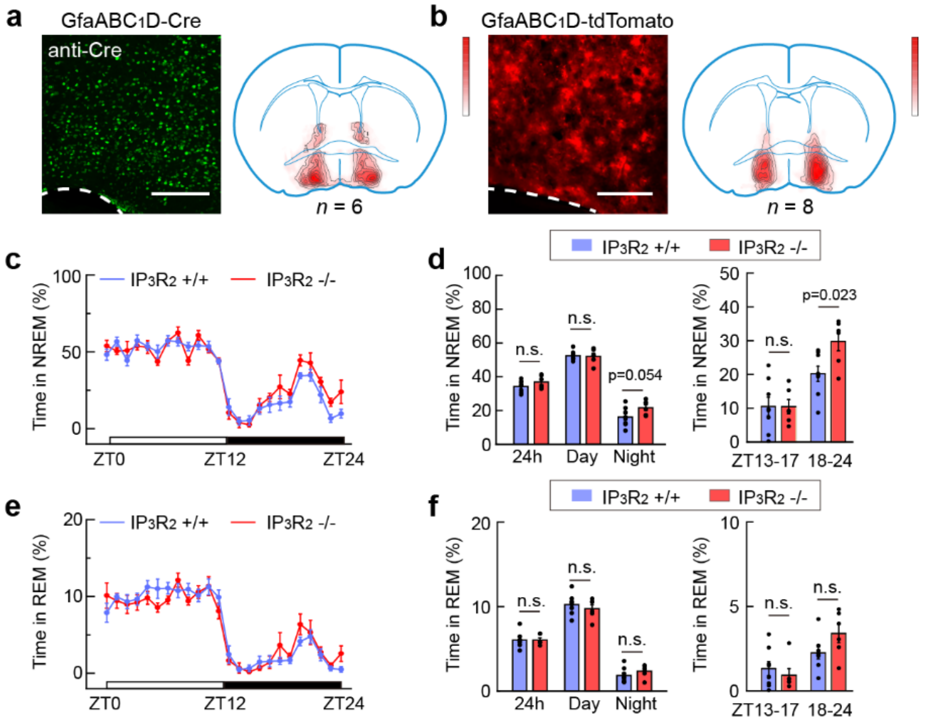

Supplementary Fig. S4 Sleep-wake modulation after IP_3R_2 knockout in BF astrocytes (related to Fig. 3)

a Fluorescence image of the BF showing the expression of the $GfaABC_1D$- Cre in the BF (left) and the normalized expression density of $GfaABC_1D$-Cre from 6 mice (right). Scale, 200 μm. **b** Same as **a**, except that $GfaABC_1D$-tdTomato was injected. **c** Circadian variation of NREM sleep in the two groups of mice. Cre group: *n* = 6 mice; tdTomato group: *n* = 8 mice. **d** Time percentage of NREM sleep in the entire 24-hour, during the day, during the night, and the early (ZT13-17) and late phase (18-24) of the night. 24h, *P* = 0.20; Day, *P* = 0.90; Night, *P* = 0.054; ZT13-17, *P* = 0.98; ZT18-24, **P* = 0.023; Student's *t*-test. **e-f** Same as **c-d**, respectively, except that data were for REM sleep. In **f**, 24h, *P* = 0.99, Student's *t*-test; Day, *P* = 0.45, Student's *t*-test; Night, *P* = 0.27; ZT13-17, *P* = 0.64, Wilcoxon rank-sum test; ZT18-24, *P* = 0.11, Student's *t*-test .

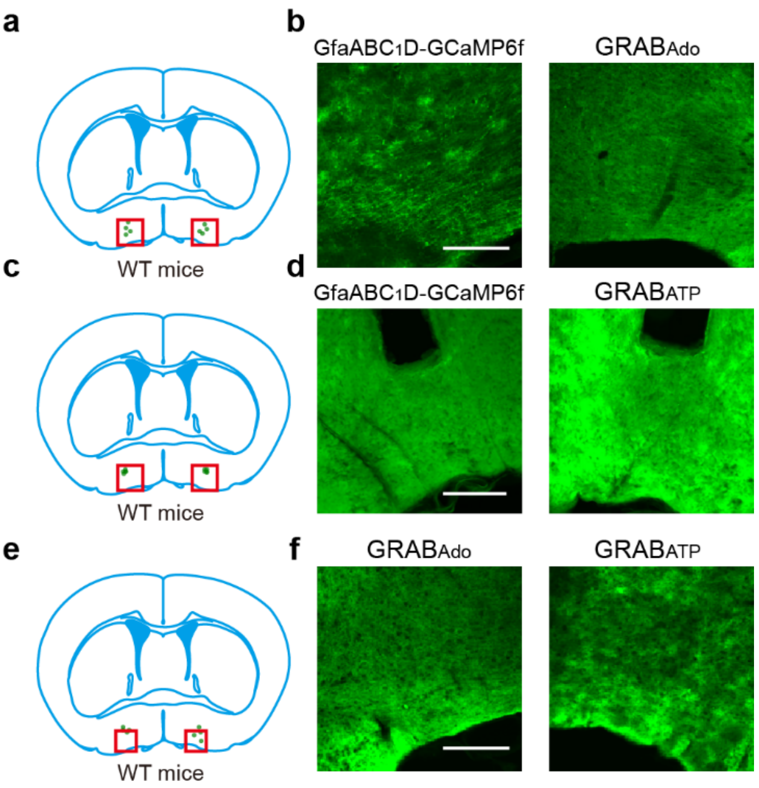

Supplementary Fig. S5　Histological verification of optic fiber position (related to Fig. 4)

a Schematic diagram summarizing placement of the optical fiber in Fig. 4**a-e**. **b** Fluorescence image of the BF showing the expression of the $GfaABC_1D$-GCaMP and hSyn-$GRAB_{Ado1.0}$. **c-f** Same as **a** and **b**, except that data were related to experiments in Fig. 4**f-j**, Fig. 4**k-o**. Scale bars represent 200 μm in all images.

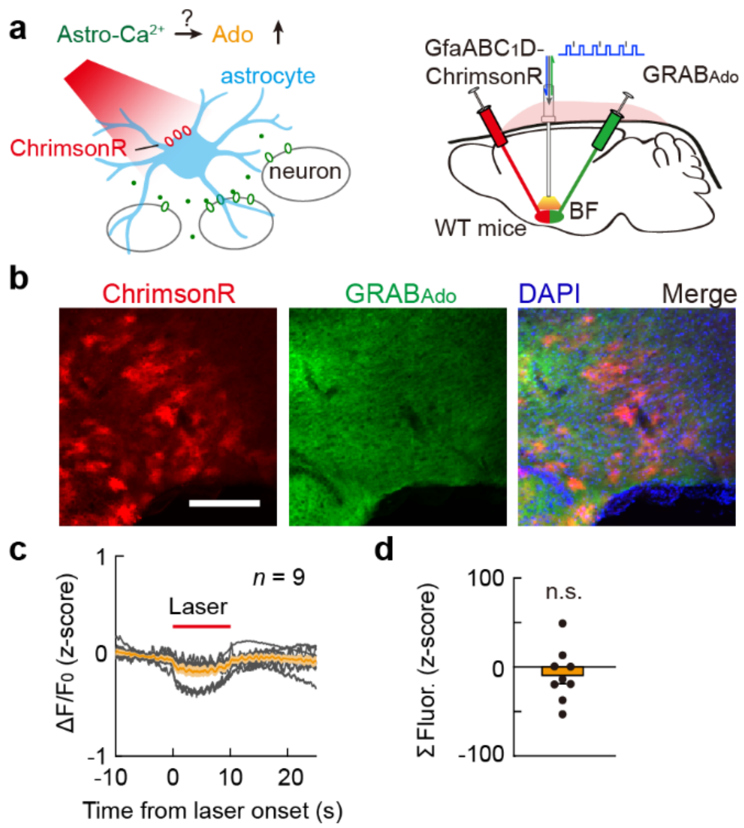

Supplementary Fig. S6 Optogenetically evoked astrocyte Ca^{2+} elevation causes no detectable adenosine increase (related to Fig. 4)

a Schematic of experiment. Fiber photometry recording of optogenetic stimulation-induced adenosine release by astrocytes. **b** Fluorescence images showing the expression of the $GfaABC_1D$-ChrimsonR and $GRAB_{Ado}$ in the BF. AAVs expressing $GfaABC_1D$-ChrimsonR (AAV5-$GfaABC_1D$-ChrimsonR) and $GRAB_{Ado}$ (AAV9-hSyn-$GRAB_{Ado1.0}$) were injected into the BF. Scale, 200 μm. **c** Laser (638 nm laser, 10 mW, 10 ms/pulse, 10 Hz for 10s)-evoked $GRAB_{Ado}$ signals. *n* = 9 mice. The red bar indicates the laser train. **d** Quantification of laser-evoked adenosine signals in **c**. Integrated signal area (*P* = 0.39, Student's *t*-test).

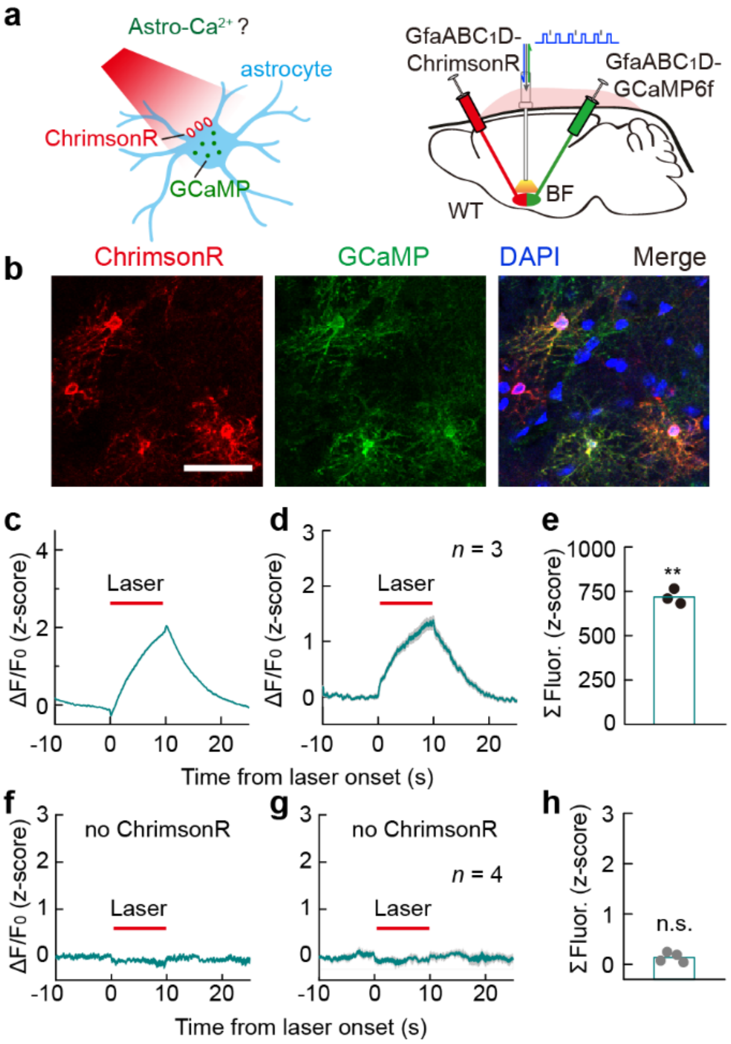

Supplementary Fig. S7 Optogenetic stimulation induces prominent astrocyte Ca^{2+} elevation (related to Fig. 4)

a Optogenetic stimulation was used to induce Ca^{2+} elevation in astrocytes. AAVs expressing $GfaABC_1D$-ChrimsonR (AAV5-$GfaABC_1D$-ChrimsonR) and $GfaABC_1D$-GCaMP (AAV5-$GfaABC_1D$-GCaMP6f) were injected into the BF. **b** Fluorescence images showing the expression of the $GfaABC_1D$-ChrimsonR and $GfaABC_1D$-GCaMP in the BF. Scale, 40 μm. **c** Laser (638 nm laser, 2 mW, 10 ms/pulse, 10 Hz for 10 s)-evoked GCaMP signals in an example experiment. The red bar indicates the laser train. **d** Group summary of laser-evoked GCaMP signals. *n* = 3 mice. **e** Quantification of laser-evoked GCaMP signals in **d**. Integrated signal area (***P* = 0.0011, Student's *t*-test). **f-h** Same as **c-e**, except that laser stimulation was applied to mice injected with AAV5-$GfaABC_1D$-

GCaMP6f only. *P* = 0.79, Student's *t*-test. Laser stimulation without ChrimsonR caused no significant change in the GCaMP signal. This experiment was used to control the non-specific effect of the laser.

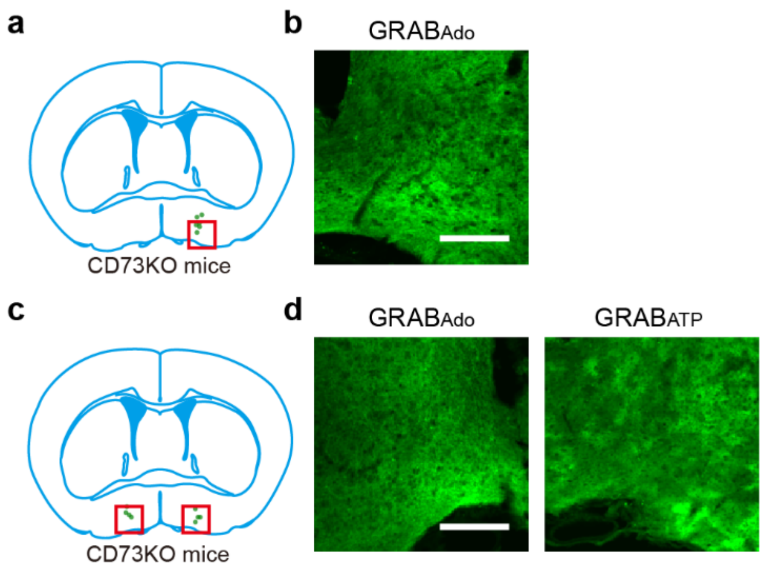

Supplementary Fig. S8 Histological verification of optic fiber position (related to Fig. 5)

a Schematic diagram summarizing placement of the optical fiber in Fig. 5**a-b**. **b** Fluorescence image of the BF showing the expression of the hSyn-GRAB$_{Ado1.0}$. **c-d** Same as **a** and **b**, except that data were related to experiments in Fig. 5**c-g**. Scale bars represent 200 μm in all images.

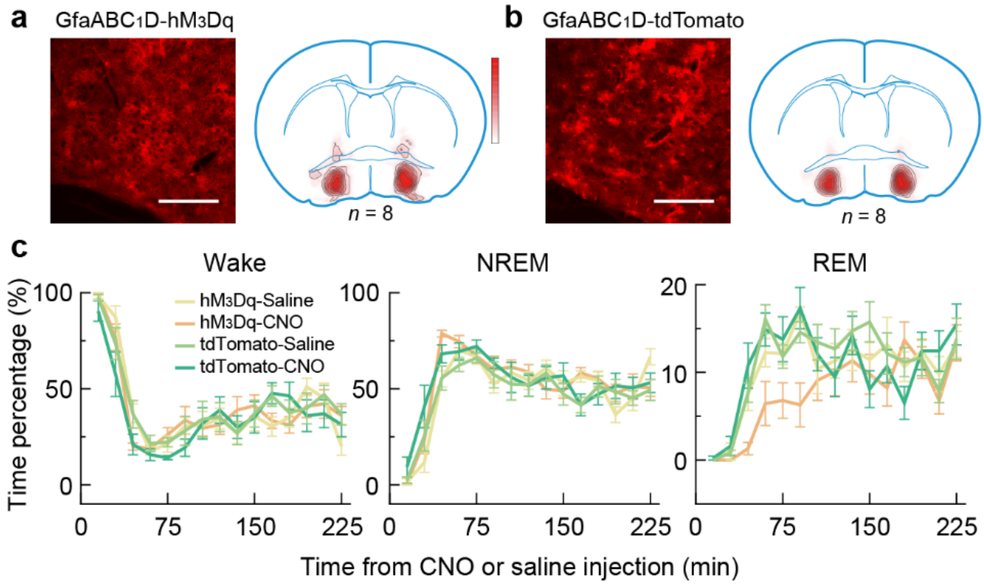

Supplementary Fig. S9 Sleep-wake modulation by chemogenetic-induced Ca^{2+} elevation in BF astrocytes in CD73KO mice (related to Fig. 5)

Same as Supplementary **Fig. S3** except that experiments were performed using CD73KO mice. In **a-b**, scale bars = 200 µm. hM3Dq group, *n* = 8 mice; tdTomato group, *n* = 8 mice.

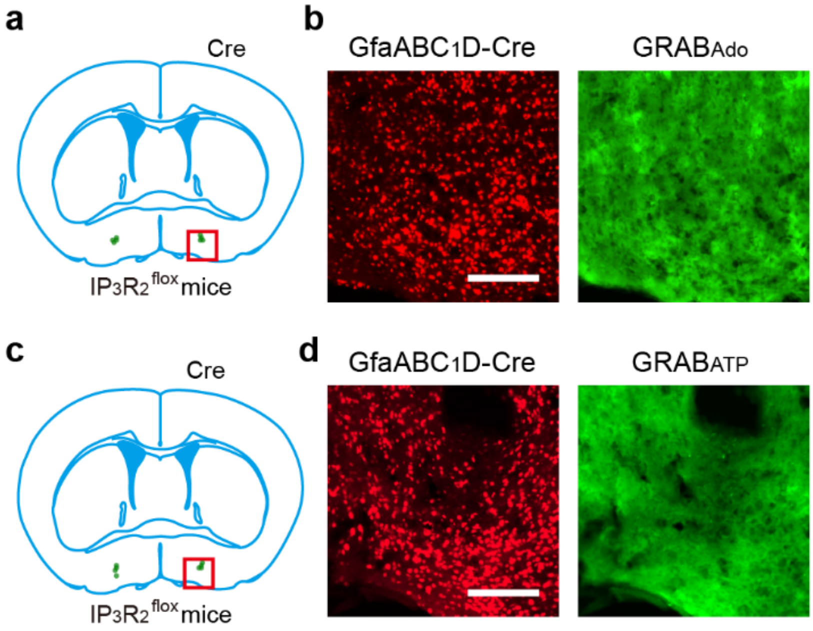

Supplementary Fig. S10 Histological verification of optic fiber position (related to Fig. 6)

a Schematic diagram summarizing placement of the optical fiber in Fig. 6**a-c**. **b** Fluorescence image of the BF showing the expression of the GfaABC$_1$D-Cre and GfaABC$_1$D-GRAB$_{Ado1.0}$. **c-d** Same as **a** and **b**, except that data were related to experiments in Fig. 6**d-f**. Scale bars represent 200 μm in all images.

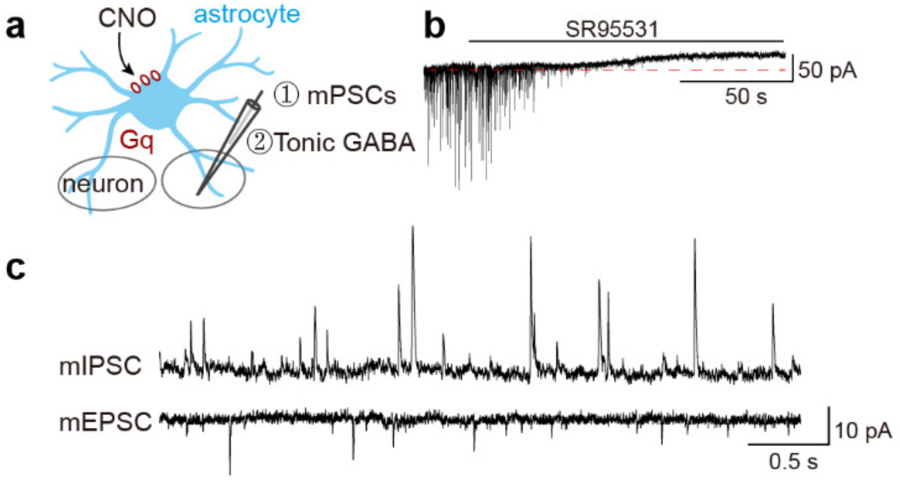

Supplementary Fig. S11 Brain slice recordings of neural network modulation induced by astrocyte activity (related to Fig. 7)

a Schematic diagram of brain slice recording. **b** Example recording of tonic GABA current. **c** Example recordings of mIPSCs (up) and mEPSCs.

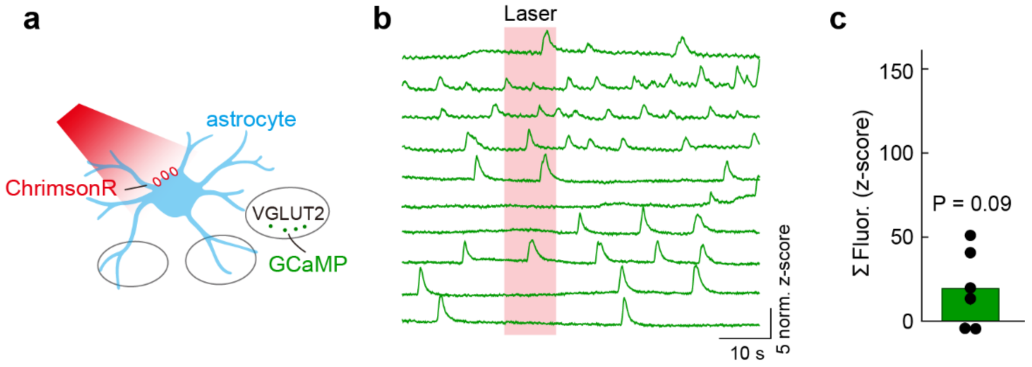

Supplementary Fig. S12 Optogenetically evoked astrocyte Ca^{2+} elevation causes no detectable Ca^{2+} increase in VGLUT2+ neurons (related to Fig. 7)

a Schematic of experiment. We activated BF astrocytes (expressing ChrimsonR) using optogenetic stimulation (638 nm laser, 10 mW, 10 ms/pulse, 10 Hz for 10s) and recorded Ca^{2+} activity of BF glutamatergic neurons (expressing GCaMP). **b** Example recording showing the Ca^{2+} activity of BF glutamatergic neurons during laser application (red shading). **c** Quantification of laser-evoked Ca^{2+} activity of BF glutamatergic neurons during laser period. Integrated signal area (P = 0.09, Student's t-test). n = 6 sessions from 4 mice.

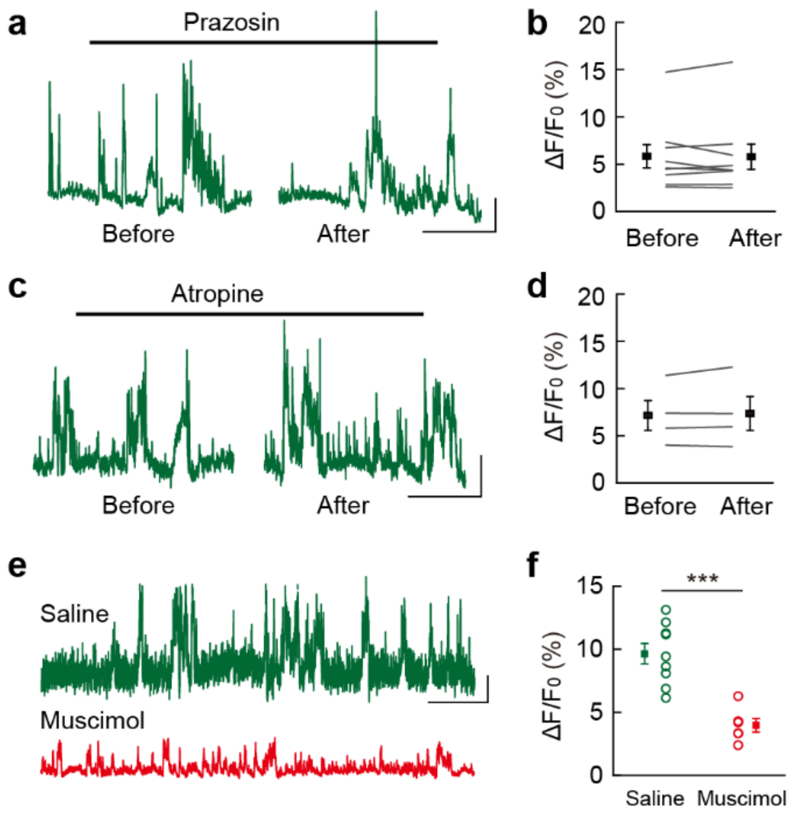

Supplementary Fig. S13 Astrocyte Ca^{2+} elevation in the BF depends on local neuronal activity (related to Fig. 7)

a Example recording of astrocyte Ca^{2+} signals before and after injection of prazosin (i.p.). Scale, 1000 s and 10%. **b** Quantification of astrocyte Ca^{2+} signals before and after prazosin injection. n = 9, P =0.91, Wilcoxon signed-rank test. **c-d** Same as **a** and **b**, respectively, except that atropine was injected. n = 4, P = 0.43, Paired t-test. **e** Example recording of astrocyte Ca^{2+} signals after infusion of muscimol (200 ng) or saline into the BF. Scale, 1000 s and 10%. **f** Quantification of astrocyte Ca^{2+} signals after infusion of muscimol or saline into the BF. n = 6 and 9 for the muscimol and saline group, respectively. ***P < 0.001, Student's t-test.

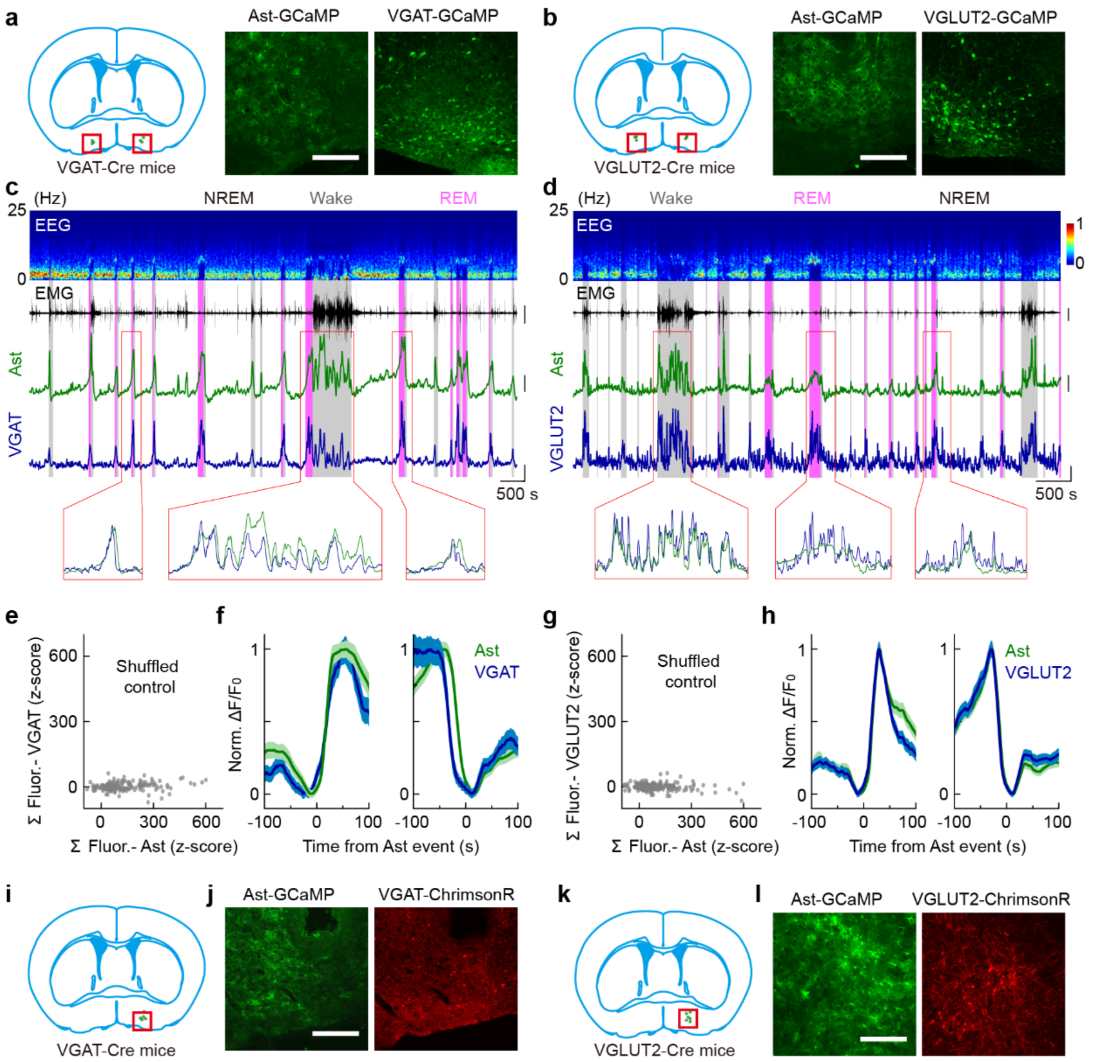

Supplementary Fig. S14 Cell type-specific neuromodulation of astrocyte Ca^{2+} elevation in the BF (related to Fig. 7)

a Schematic diagram summarizing the optical fiber placement in mice expressing $GfaABC_1D$-GCaMP and DIO-GCaMP in the BF of VGAT-Cre mice (left), and fluorescence images of the BF (red boxes in coronal diagram) showing the expression of the $GfaABC_1D$-GCaMP and DIO-GCaMP. Scale, 200 μm. **b** Same as **a**, except that experiments were performed in VGLUT2-Cre mice. **c** Top to bottom, EEG power spectrogram, EMG (scale, 1 mV), GCaMP fluorescence of astrocytes in the BF (scale, 1 z-score), and GCaMP fluorescence of the BF VGAT+ neurons (scale, 1 z-score). **d** Same as **c**, except that recording was from BF VGLUT2+ neurons. **e** Correlation between the size of GCaMP events from BF astrocytes and BF VGAT+ neurons. Signals from

BF VGAT+ neurons were temporally shuffled randomly. Pearson's r = -0.13. **f** Time course of the GCaMP events from BF astrocytes and BF VGAT+ neurons aligned to the onset (left) or offset (right) of the GCaMP events from BF astrocytes. **g-h** Same as **e** and **f**, respectively, except that GCaMP events from BF astrocytes and BF VGLUT2+ neurons were compared. Pearson's r = 0.10 in **g**. **i** Schematic diagram summarizing the optical fiber placement in VGAT-Cre mice expressing DIO-ChrimsonR and GfaABC$_1$D-GCaMP. **j** Fluorescence images of the BF (red box in coronal diagram) showing the expression of the DIO-ChrimsonR and GfaABC$_1$D-GCaMP. Scale, 200 μm. **k-l** Same as **i** and **j**, respectively, except that experiments were performed in VGLUT2-Cre mice.

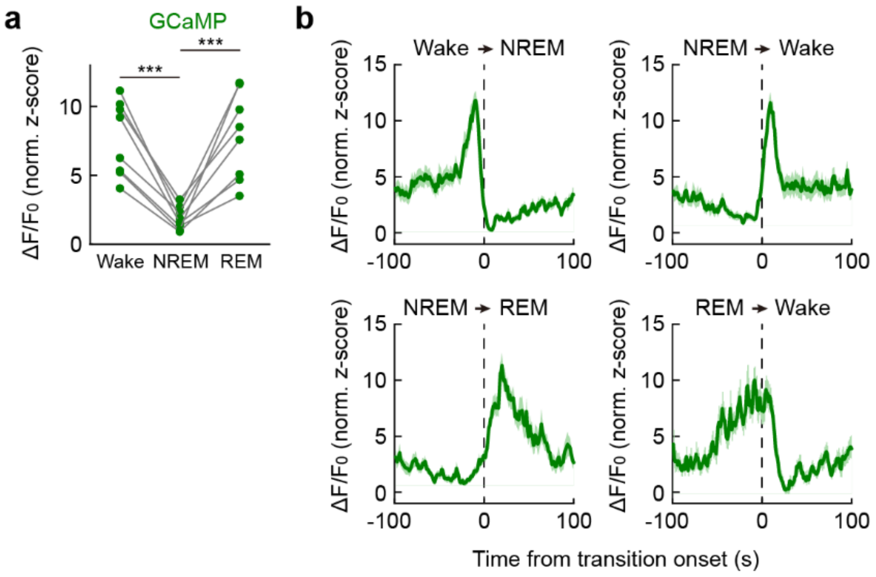

Supplementary Fig. S15 Brain state-dependent astrocyte Ca^{2+} activity in the LC/SLD during the sleep-wake cycle (related to Fig. 8)

a GCaMP fluorescence in different brain states. Each line represents data from one recording. *n* = 8 sessions from 3 mice; Wake vs. NREM: ****P* < 0.001; REM vs. NREM: ****P* < 0.001; Paired *t*-test. **b** GCaMP signal during brain state transitions. The vertical dashed lines represent the transition time. *n* = 95, 70, 71, and 46 events (in 3 mice) for 'Wake to NREM', 'NREM to Wake', 'NREM to REM', and 'REM to Wake', respectively.

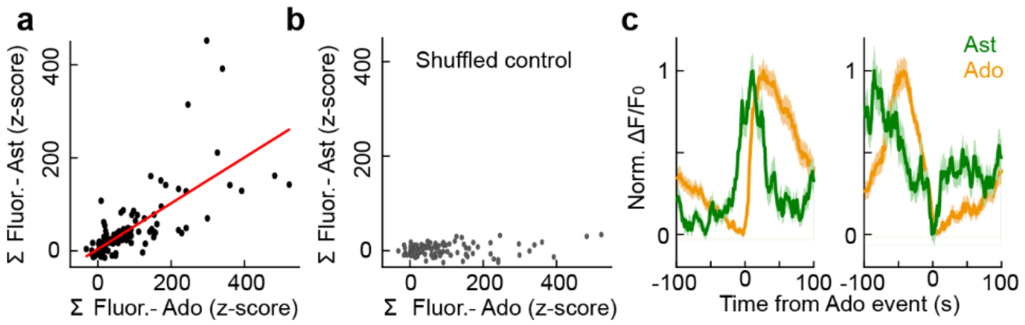

Supplementary Fig. S16 Astrocyte Ca^{2+} elevation in the LC/SLD is highly correlated with the dynamics of extracellular adenosine (related to Fig. 8)

a Correlation between the size of GCaMP and $GRAB_{Ado}$ events. The red line represents a linear fit. *n* = 130 events from 8 recordings in 3 mice. Pearson's r = 0.72, ****P* < 0.001. **b** Same as in **c** after the GCaMP signal was randomly shuffled. Pearson's r = 0.23. **c** Time course of the GCaMP and $GRAB_{Ado}$ signal aligned to the onset (left) or offset (right) of the $GRAB_{Ado}$ events.

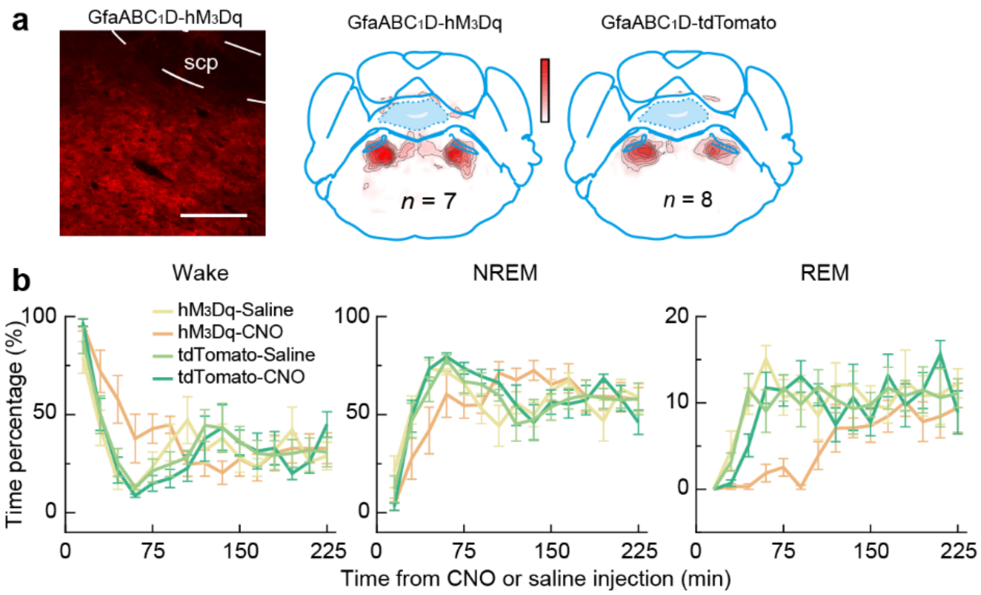

Supplementary Fig. S17 Sleep-wake modulation by chemogenetic-induced ca^{2+} elevation in LC/SLD astrocytes in WT mice (related to Fig.8)

Same as Supplementary information **Fig. S3** except that experiments were performed in LC/SLD. In **a**, scale bar = 200 μm. hM3Dq group, *n* = 7 mice; tdTomato group, *n* = 8 mice.

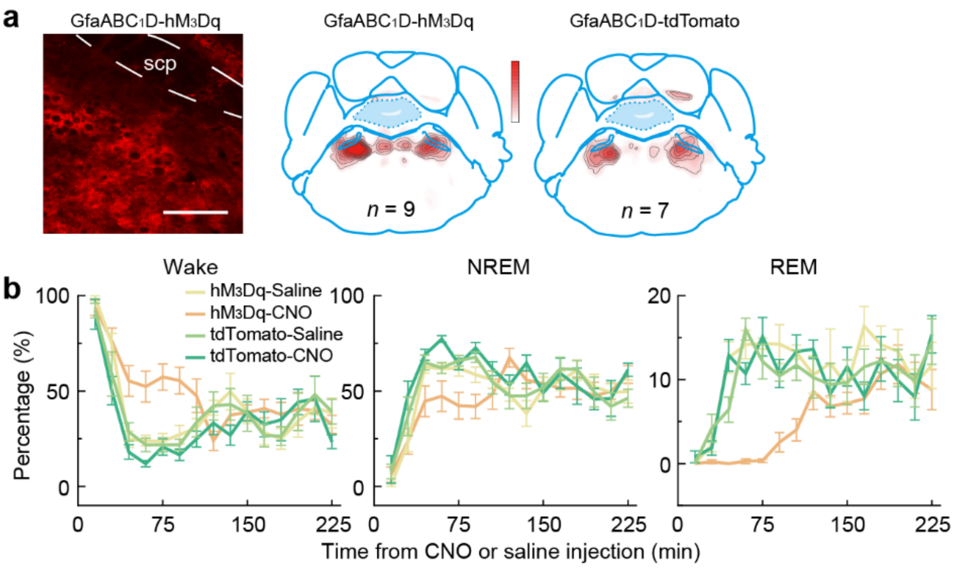

Supplementary Fig. S18 Sleep-Wake modulation by chemogenetic-induced Ca^{2+} elevation in LC/SLD astrocytes in CD73KO mice (related to Fig. 8)

Same as Supplementary information **Fig. S17** except that experiments were performed using CD73KO. In **a**, scale bar = 200 μm. hM_3Dq group, *n* = 9 mice; tdTomato group, *n* = 7 mice.

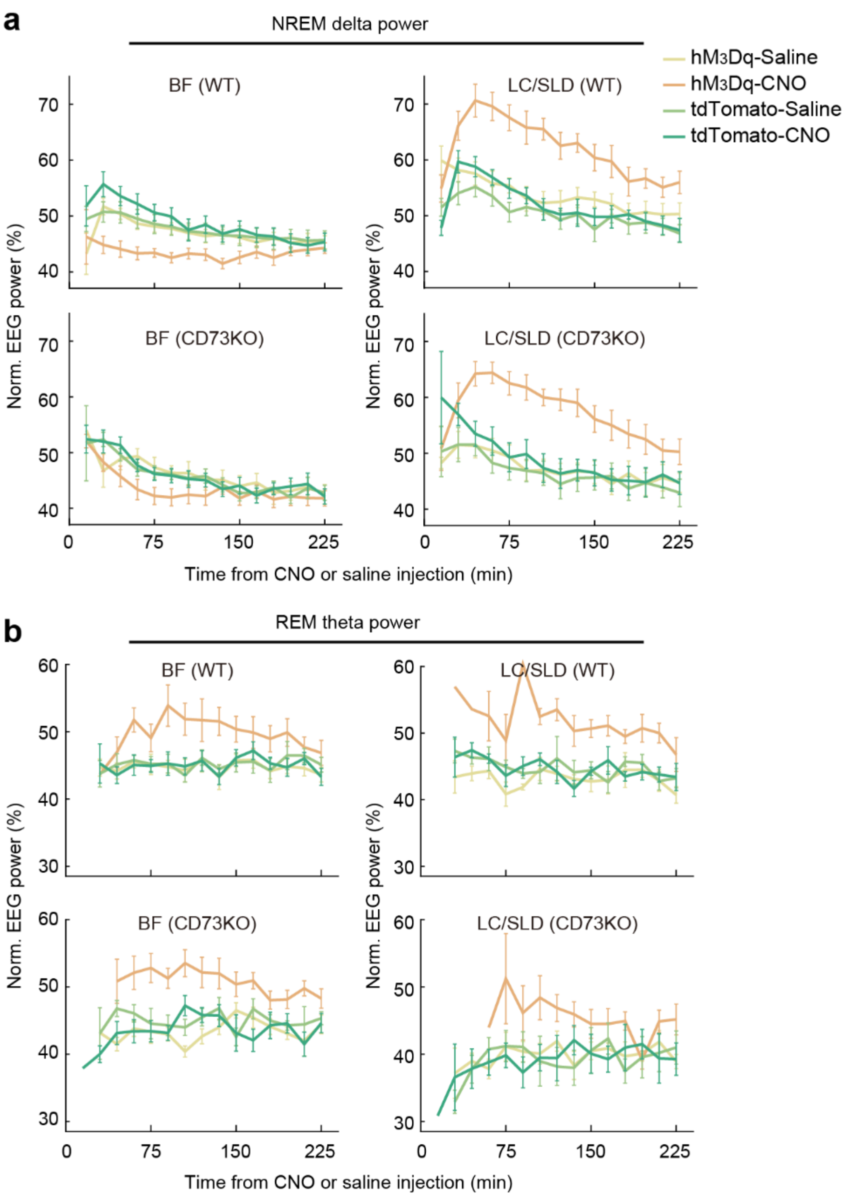

Supplementary Fig. S19 Time course of EEG power after CNO injection (related to Fig.8)

a Time course of NREM EEG delta power after CNO injection in the four sets of experiments. **b** Time course of REM EEG theta power after CNO injection in the four sets of experiments.
